# Pharmacological chromatin remodeling enhances response to estrogen therapy in ER ^+^ breast cancer

**DOI:** 10.1002/1878-0261.70307

**Published:** 2026-07-17

**Authors:** Anneka L. Johnson Thomas, Barbara Karakyriakou, Patricia C. Muskus, Alyssa M. Roberts, Justin Rendleman, Alexa Warren, Hanxu Lu, Kimberly A. Birmingham, Malone K. Friedman, Abigail E. Goen, Huijuan Yang, Owen M. Wilkins, Fred W. Kolling, Eugene Demidenko, Julie M. Jorns, Brock C. Christensen, Todd W. Miller

**Affiliations:** ^1^ Department of Molecular and Systems Biology Dartmouth Geisel School of Medicine Lebanon NH USA; ^2^ Department of Pharmacology and Toxicology Medical College of Wisconsin Milwaukee WI USA; ^3^ Department of Epidemiology, Geisel School of Medicine Dartmouth College Lebanon NH USA; ^4^ Laboratory of Genome Architecture and Dynamics The Rockefeller University New York NY USA; ^5^ Department of Biomedical Data Science Geisel School of Medicine at Dartmouth Lebanon NH USA; ^6^ Dartmouth Cancer Center Dartmouth Geisel School of Medicine Hanover NH USA; ^7^ Department of Biostatistics Dartmouth Geisel School of Medicine Lebanon NH USA; ^8^ Department of Pathology Medical College of Wisconsin Milwaukee WI USA

**Keywords:** breast cancer, endocrine resistance, estrogen therapy

## Abstract

Estrogen therapy elicits clinical benefit in ~ 30% of patients with endocrine‐resistant estrogen receptor (ER)‐positive breast cancer, but its mechanism of action and strategies to increase efficacy remain unclear. Estrogen therapy can induce ER transcriptional hyperactivation and DNA damage; we postulated that such damage could be exacerbated by epigenetic dysregulation via inhibition of histone deacetylases (HDACi). We evaluated the effects of 17b‐estradiol and HDACi in three types of ER^+^ breast cancer models: Cells adapted to growth following long‐term estrogen deprivation; cells engineered to overexpress exogenous ER that confers endocrine resistance; mice bearing endocrine‐resistant patient‐derived xenografts. Assay endpoints included apoptosis, growth, DNA damage, histone post‐translational modification, levels of ER‐regulated transcripts and encoded proteins, cell cycle and replication status, and genome‐wide chromatin accessibility, ER binding, and transcriptional profiles. Entinostat treatment increased histone acetylation and chromatin accessibility. Combination treatment with E2 and entinostat inhibited cell growth and induced apoptosis in ER‐overexpressing models. E2 and entinostat induced DNA damage as single agents and in combination. These agents synergized against tumor models, offering HDACi as a strategy to enhance efficacy of estrogen therapy.

## Graphical abstract

Estrogen therapy elicits clinical benefit in ~ 30% of patients with endocrine‐resistant estrogen receptor (ER)‐positive breast cancer. Based on findings that ER transcriptional activation underlies response to estrogen therapy, we tested the effects of epigenetic dysregulation via pharmacological inhibition of histone deacetylases (HDACi). Combination treatment with estrogen (17b‐estradiol) and HDACi inhibited cell growth, induced apoptosis, and synergized against tumor models.

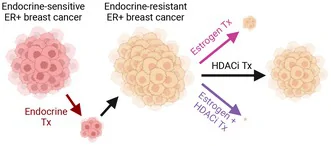

**Abbreviations**
BC: breast cancer
DCC‐FBS: dextran/charcoal‐treated FBS
DMEM: Dulbecco's modified Eagle's medium
E2: 17b‐estradiol
ER: estrogen receptor alpha
FBS: fetal bovine serum
HDAC: histone deacetylase
HDACi: histone deacetylase inhibition/inhibitor
HER2: human epidermal growth factor receptor 2
NSG^−^
: NOD‐scid/IL2Rγ^−/−^
PDX: patient‐derived xenograft

## 1 Introduction

The most prevalent subtype of breast cancer (BC) is estrogen receptor‐positive/human epidermal growth factor receptor 2‐negative (ER^+^/HER2^−^) disease, making up 2/3 of BC cases (~ 200 000 new diagnoses annually in the U.S.). The transcriptional profile promoted by ER signaling canonically drives BC cell growth and proliferation. Conventional endocrine therapies interrupt this process by inhibiting estrogen biosynthesis (with aromatase inhibitors, AIs) or antagonizing ER (with tamoxifen) [1, 2]. Despite adjuvant treatment with endocrine therapy, ~ 1/3 of patients experience BC recurrence that is typically metastatic and ultimately fatal [3, 4, 5]. Thus, there remains a need to improve therapy for endocrine‐resistant disease.

Estrogen therapy was observed to be effective against BC in the 1940s [6]. Estrogen therapy with diethylstilbestrol was as effective as tamoxifen at increasing progression‐free survival (PFS) [7]. Estrogen therapy elicits clinical benefit in ~ 30% of patients with endocrine‐resistant ER^+^ BC [8, 9, 10, 11, 12, 13]. However, the use of estrogen therapy is limited due to the paradox of treating BC with estrogen (which promotes growth of treatment‐naïve BC), lack of mechanistic understanding of efficacy, and absence of rational combination therapies to enhance efficacy.

Two case reports described response to 17b‐estradiol (E2) in patients with *ESR1* (ER)‐amplified ER^+^ advanced breast tumors [14, 15]. The *ESR1‐*amplified WHIM16 patient‐derived xenograft (PDX) model grows in ovariectomized mice, regresses in response to E2 treatment, and is growth‐suppressed by the anti‐estrogen fulvestrant [15, 16, 17]. We showed ER overexpression can confer resistance to estrogen deprivation through ER activation in ER^+^ breast cancer cells and xenografts grown in mice. However, ER overexpression and the associated high levels of ER transcriptional activation converted E2 from a growth‐promoter to a growth‐suppressor [17]. We also described induction of DNA damage by ER transcriptional (re‐)activation with E2 or anti‐estrogen withdrawal in endocrine‐resistant ER^+^ BC models [17, 18]. We postulated that pharmacological inhibition of histone deacetylases (HDACi), which increases chromatin accessibility [19], would promote E2‐induced ER‐DNA binding and transcription with consequent DNA damage in endocrine‐resistant BC cells. HDACi have been approved clinically to treat hematological malignancies, but effective strategies for use in solid tumors remain unproven. HDACs are differentially expressed across BC subtypes, but ER^+^ tumors show relatively higher HDAC1 levels with 38% of tumors considered ‘high‐expressing’ [20]. Clinical testing of HDACi for advanced ER^+^ BC yielded mixed results. The HDACi entinostat and tucidinostat were tested in combination with the AI exemestane in Phase III clinical trials: HDACi cotreatment increased PFS compared to placebo [21, 22]. In contrast, another Phase III trial showed entinostat plus exemestane did not change PFS or overall survival compared to exemestane/placebo [23]. While the ability of endocrine therapy to synergize with HDACi has shown mixed results, it has yet to be described whether HDACi can modulate the effect of estrogen therapy in endocrine‐resistant BC. We therefore undertook this study to determine the effects of HDACi on response to E2 therapy in preclinical models of ER^+^ BC.

## 2 Materials and methods

### 2.1 Cell culture

HCC‐1428 cells (RRID: CVCL_1252) and MDA‐MB‐415 cells (RRID: CVCL_0621) (ATCC) were cultured in DMEM with 10% FBS (R&D Biosystems, Minneapolis, MN, USA). HCC‐1428/LTED cells were previously derived through long‐term (~ 1 year) maintenance in hormone‐depleted (HD) conditions via phenol red‐free DMEM with 10% dextran/charcoal‐treated FBS (DCC‐FBS; R&D Biosystems) as described [24]. MDA‐MB‐415 cells were previously engineered to stably overexpress ESR1 (or luciferase control) with a lentiviral vector; MDA‐MB‐415/ESR1 cells were maintained in HD medium [17]. Cell lines were authenticated by STR genotyping, confirmed to be mycoplasma‐free, and used for experimentation within 4 months of culture.

### 2.2 Mouse studies

Mouse studies were approved by the IACUC review boards at Dartmouth College (#00002144) and the Medical College of Wisconsin (#8300). Female NOD‐scid/IL2Rγ^−/−^ (NSG) mice (4–5 weeks old) were obtained from an in‐house colony at Dartmouth College or purchased from The Jackson Laboratory and ovariectomized (ovx) to reduce circulating estrogen levels [25]. The CTG‐3346 PDX model was previously established from a recurrent breast tumor biopsy [18]. The WHIM16 PDX model was obtained from Washington University [15]. Both models were established from metastatic ER^+^ BC specimens from patients with endocrine‐resistant disease. Fresh PDX tumor fragments (~ 8 mm^3^) from a donor mouse were serially transplanted subcutaneously into ovx mice at 4–6 weeks of age. Calipers were used to measure tumor dimensions twice weekly blind of treatment group; volume was calculated as (length × width^2^)/2. When volume reached ≥ 200 mm^3^, a mouse was randomized to drug treatment: vehicle (sulfobutylether‐b‐cyclodextrin (SbC) in 20% saline); E2 (s.c. wax pellet, 1 mg [26]), entinostat (dissolved in DMSO and diluted in SbC, 10 mg·kg^−1^·day^−1^, p.o. 5 days·week^−1^; MedChemExpress #HY‐12163), or the combination. Entinostat was administered for 7.5 weeks. E2 pellets remained in mice for the duration of study.

Molecular analysis of CTG‐3346 tumors was done on 200‐mm^3^ tumor tissues from mice treated for 3 days ± E2 (100 μg·day^−1^, p.o., BID, which elicits antitumor effects similarly to s.c. pellets [17]) and entinostat. On Day 3 of drug treatment, mice were dosed with drug, 1 h later injected i.p. with EdU (30 mg·kg^−1^ in 100 μL PBS), and tumors harvested 3 h later post‐EdU. WHIM16 tumor‐bearing mice were treated with vehicle or entinostat for 14 days, and cotreated ± E2 (100 μg·day^−1^, p.o., BID) for the final 3 days. Tumor tissues were snap‐frozen or formalin‐fixed and paraffin‐embedded (FFPE).

### 2.3 Immunoblotting

All reagents were obtained from Sigma unless otherwise specified. Cell lysis was performed with RIPA buffer (20 mmol·L^−1^ Tris, pH 7.4, 1% NP‐40, 10% glycerol, 1 mmol·L^−1^ EGTA, 1 mmol·L^−1^ EDTA, 5 mmol·L^−1^ NAPPi, 10 mmol·L^−1^ Na b‐glycerophosphate, 50 mmol·L^−1^ NaF, 150 mmol·L^−1^ NaCl) with 1 nmol·L^−1^ Na_3_VO_4_ (New England Biolabs, Ipswich, MA, USA) and 1× Protease Inhibitor Cocktail (Millipore‐Sigma, Burlington, MA, USA) on ice for 10 min. Snap‐frozen tumor chunks were thawed/homogenized and lysed in the same RIPA buffer. Lysates were sonicated for 15 s on ice, and centrifuged at 18 000 ***g*** for 10 min at 4 °C. BCA assay (Pierce) was used to quantify and standardize protein concentrations across samples. Lysates were reduced and denatured using LDS sample buffer (GenScript, Piscataway, NJ, USA) and 1.25% b‐mercaptoethanol. Proteins were separated by SDS/PAGE and transferred to a nitrocellulose membrane. Membrane was stained with Ponceau S to enable visual confirmation of protein transfer. Membranes were blocked with 5% BSA in TBS‐T and probed with primary antibodies against acetyl‐histone H3 Lys27 (Cell Signaling Technology, Danvers, MA, USA, #8173), acetyl‐histone H3 Lys9 (Cell Signaling Technology #9649), IRS‐1 (Cell Signaling Technology #3407), full‐length and cleaved PARP (Cell Signaling Technology #9532), progesterone receptor A/B (Cell Signaling Technology #3153), IGF‐1Rb (Cell Signaling Technology #3027), TFF1 (Cell Signaling Technology #15571), ER (Santa Cruz Biotechnology, Dallas, TX, USA, #sc‐8002), vinculin (Cell Signaling Technology #13901), or b‐Actin (Cell Signaling Technology #3700) in blocking solution overnight at 4 °C. Species‐specific secondary antibodies conjugated to horseradish peroxidase (HRP) (GE Healthcare, Chicago, IL, USA) were utilized to detect primary antibodies, and HRP was detected using ECL substrate (Thermo Scientific, Waltham, MA, USA, #32106). Chemiluminescent signal was measured using ImageQuant 800 (Cytiva Amersham, Marlborough, MA, USA) or ChemiDoc MP (Bio‐Rad, Hercules, CA, USA).

### 2.4 Assay for transposase‐accessible chromatin with sequencing (ATAC‐seq)

HCC‐1428/LTED cells in 6‐well plates were treated ±500 nmol·L^−1^ entinostat for 1 or 3 days in triplicate wells (500 000–1 000 000 cells per well). Cells were processed using an ATAC‐seq kit (Active Motif #53150), which included cell lysis, nuclei isolation, tagmentation reaction, DNA purification, and library preparation. DNA sequencing was performed by the Genomics and Molecular Biology Shared Resource (Dartmouth College) on an Illumina NextSeq2000 to obtain 50‐bp paired‐end reads.

Raw FASTQ files were processed in r software by Nextflow nf‐core/atacseq pipeline (version 2.1.2) with default settings [27]. Sequencing reads were aligned to GRCh38 human reference genome with bowtie2 [28]. Quality control was performed according to the ENCODE ATAC‐seq pipeline, including assessment of sequencing quality, alignment metrics, fragment size distribution, and enrichment of reads at transcription start sites (TSS). Peak calling was run under narrow peak parameter by MACS2 [29] with a threshold of FDR < 0.01. Peaks and genome‐wide chromatin accessibility signal tracks were generated following the ENCODE ATAC‐seq pipeline using filtered, duplicate‐removed, and Tn5‐shifted alignments, and normalized to reads per genomic content. ATAC‐seq signal data were further processed using deeptools' (v3.5.6) computeMatrix function [30] to calculate signal scores across genomic regions and produce heatmap visualizations. The reference‐point mode was used with the center of each peak as the reference, and a 3‐kb window was set upstream and downstream (−3000 to +3000 bp) to capture surrounding signal distribution. To identify genomic regions significantly altered by treatment with entinostat, differential accessibility analysis was performed using the deseq2 (v1.50.2) package in r (v4.5.1). Read counts over identified peaks were calculated using the featureCounts function, with BAM files from each replicate and a combined peak set derived from both entinostat‐treated and vehicle‐treated samples, using the bedtools (v2.30.0) merge function, as input. The resulting count matrix was imported into a DESeqDataSet object and analyzed with the DESeq function to compute log_2_ fold changes and significance estimates. For each pairwise comparison between entinostat‐treated samples and vehicle‐treated controls, differential accessibility results included log_2_ fold changes, raw *P*‐values, and Benjamini–Hochberg‐adjusted *P*‐values for each peak.

To test for overrepresentation of transcription factor motifs within the accessible regions altered by entinostat treatment, motif enrichment analysis was performed using the chromvar (v1.32.0) r package. Prior to analysis, all peak regions were resized to 500‐bp bins centered on peak summits to ensure uniformity. Motif scanning and enrichment testing were conducted using the JASPAR2024 motif collection.

### 2.5 Growth assays

Prior to seeding, parental cells were HD for 3 days to establish basal ER activity. Cells were then seeded in HD medium in triplicate at 7000 cells per well in a 12‐well plate and treated as indicated for up to 4 weeks or until control wells reach ~ 70% confluency, whichever came first. Cells were fixed and stained with 0.5% crystal violet in 20% methanol for 10 min. Unbound dye was rinsed out with water, and plates were dried overnight. Plates were scanned using the LiCor Odyssey M. Relative levels of staining were measured using ImageJ (RRID:SCR_003070).

### 2.6 RT‐qPCR

Parental cells were pretreated with HD for 3 days prior to seeding at 700 000 cells per well in HD medium in 6‐well plates. Cells were treated with HD ± 1 nm E2 and 500 nm entinostat for 24 h. RNA was harvested using RNeasy Plus Mini Kit (Qiagen, Germantown, MD, USA, #1062832) and quantified by NanoDrop. RNA concentrations were normalized across samples. RNA was reverse‐transcribed using iScript cDNA Synthesis kit (Bio‐Rad #1708890). Real‐time quantitative PCR (qPCR) reaction was prepared in triplicate using iQ SYBR Green Supermix (Bio‐Rad #1708880) and primers for AREG, PDZK1, TFF1, PR, ESR1 (sequences listed in Table S1.). A Bio‐Rad CFX96 thermocycler was utilized for qPCR and data collection. Data were analyzed utilizing ΔΔ*C*_T_ method.

### 2.7 Cleavage under targets & release using nuclease (CUT&RUN)

HCC‐1428/LTED cells were treated in duplicate ±500 nm entinostat (or vehicle) for 24 h and ± 1 nm E2 (or vehicle) for the final 1.5 h. Samples were processed for CUT&RUN and library prep following the manufacturer's instructions for the CUTANA ChIC/CUT&RUN kit (EpiCypher, Durham, NC, USA, #14‐1048) and the CUTANA CUT&RUN Library prep kit (EpiCypher #14‐1001). Libraries were sequenced by Novogene using a NovaSeq X Plus Series to acquire 150‐bp paired‐end reads.

#### 2.7.1 Alignment

Adapter sequences were trimmed with cutadapt 1.18 (−e 0.1 ‐q 30 ‐m 5), and reads were aligned to the human genome (hg38/GRCh38) with bowtie2 v2.3.4.3 (−k 4 ‐p 6 ‐X2000 ‐‐local –seed [28]). We marked duplicates with picardtools v2.20.3‐0 and removed nonprimary alignments, then passed only properly paired reads to downstream analysis by filtering appropriate flags with samtools 1.9 (‐F 1804 ‐f 2).

#### 2.7.2 Peak calling

Aligned reads were used to call peaks with macs2 v2.2.4 (−f BAM ‐g hs –keep‐dup auto –seed [28]) using a vehicle‐treated IgG control as the control bam (−c); the minimum FDR cutoff for peak detection was 0.05. A consensus peak set was generated by combining all narrow peak calls and merging overlapping peaks with bedtools merge v2.29.0 (−d 10) and filtered against the ENCODE exclusion list (https://www.encodeproject.org/files/ENCFF940NTE/). Peaks were assigned to their genomic annotation and the nearest gene using the ChIPseeker package (v1.22.0), based on known gene transcript locations annotated by UCSC in hg38 (TxDb.Hsapiens.UCSC.hg38.knownGene 3.10.0) with promoters being defined by ±3 kbp from transcription start sites. To assign peaks to their nearest gene from to compare RNA expression patterns, the median distance of a peak to the transcription start site was ~ 20 kbp with a maximum distance of 3.9 Mbp. Insertion counts within peaks were generated with the Rsubread 1.32.4 featureCounts function (allowMultiOverlap = TRUE, isPairedEnd = TRUE, primaryOnly = TRUE).

#### 2.7.3 Differential binding analysis

Count normalization and differential peak analysis was performed using DESeq2 1.34.0. Differential peak accessibility was determined using Wald tests with three contrasts: E2 treatment vs. Vehicle; E2/Entinostat vs. E2; E2/Entinostat vs. Entinostat. Tests included three replicates per condition. To compare the overlap of ER peaks between treatments, peaks identified within each condition were merged, intersected with the consensus peak set, and counted.

#### 2.7.4 Motif enrichment analysis

CUT&RUN peak coordinates were obtained from peak‐calling output and formatted as genomic intervals. Peak sequences were extracted from the hg38 reference genome using the BSgenome.Hsapiens.UCSC.hg38 (v1.4.5) package. Only peaks mapping to standard chromosomes were retained for downstream analyses. Estrogen response elements (EREs) were identified by scanning peak sequences with position weight matrices (PWMs) for ESR1 (MA0112.4) and ESR2 (MA0258.2) obtained from the JASPAR 2024 (v0.99.7) CORE vertebrate database. Motif scanning was performed using Biostrings::matchPWM (v2.78.0) on forward and reverse strands. Motif matches were defined using a minimum relative PWM score threshold of 80%, unless otherwise specified. The proportion of CUT&RUN peaks containing at least one ER‐alpha/ER‐beta (ESR1/ESR2) motif match was compared to a background expectation derived from matched random genomic regions. Random regions were generated by sampling genomic intervals with the same length and chromosome distribution as the observed CUT&RUN peaks, ensuring all regions fell within chromosome boundaries. For each randomization, motif scanning was performed using the same PWMs and score thresholds as applied to observed peaks. Enrichment was evaluated using Fisher's exact test by comparing the number of motif‐containing peaks in the observed data to the number identified in the matched random background.

### 2.8 RNA sequencing (RNA‐seq)

HCC‐1428 and HCC1‐1428/LTED cells were maintained in triplicate in HD medium for 3 days, and then treated ±500 nm entinostat for 1, 3, or 7 days, and ±1 nm E2 for the final 1 day, as single agents and in combination. RNA was extracted using RNeasy Plus Mini Kit and stored at −80 °C. Library preparation and sequencing were performed by Novogene with an Illumina NovaSeq X‐Plus to obtain 150‐bp paired‐end reads.

Raw FASTQ files were processed using the nf‐core/rnaseq pipeline (version 3.18.0) with default parameters. Sequencing reads were aligned to the GRCh38 human reference genome using STAR, with transcript‐level quantification performed by Salmon in alignment‐based mode. In R, transcript‐level abundance estimates were imported using tximport (version 1.34.0). Differential gene expression was determined using DESeq2 package (version 1.46.0) utilizing Benjamini–Hochberg adjustment for multiple testing correction. Variance stabilizing was performed utilizing regularized log transformation (rlog) from the DESeq2 package. Variance stabilized object was subsequently used for principal component analysis (PCA) and volcano plots. PCA was used under default settings to visualize variation between conditions. Coefficient of variation (CV) was calculated for each gene across all samples. The top 500 genes with the highest CV ≥ 0.03 were used for heatmap visualization. Expression values of those 500 genes were scaled (*Z*‐score) across samples, and a heatmap was generated using complexheatmap package (version 2.22.0). Gene set enrichment analysis (GSEA) was performed with clusterprofiler package (version 4.14.6) with Hallmark gene sets obtained from human msigdbr database (version 10.0.1).

Transcriptomic changes induced by drug treatments were visualized using a heatmap of genes associated with regions of ER binding established by the CUT&RUN analysis. Transcript abundance data (gene × sample matrix) were imported into r (v4.4.1) and merged with the list of unique gene symbols identified from CUT&RUN peak overlaps. Expression values were subset to this gene list, and rows with zero or invariant expression across samples were removed. Two sample groups were defined corresponding to the 1‐ and 3‐day treatment comparisons (Vehicle, 1 nm E2, 500 nm entinostat, and E2 + entinostat), and expression matrices were log_2_‐transformed. For heatmap visualization, values were scaled by row (*z*‐score) to highlight relative up‐ and down‐regulation across treatment groups. Hierarchical clustering was performed using Euclidean distance and complete linkage for both genes and samples. Heatmaps were generated using the pheatmap r package (v1.0.12).

For tumor RNA‐seq analysis, extracted RNA was used for RNA sequencing by Plasmidsaurus using Illumina Sequencing Technology. Data were bioinformatically analyzed as follows. FastQ generation and demux with bcl convert v4.3.6 and fqtk v0.3.1. Read‐filtering using fastp v0.24.0: poly‐X tail trimming, 3′ quality‐based tail trimming, a minimum Phred quality score of 15, and a minimum length requirement of 50 bp. Alignment to the appropriate reference genome using star aligner v2.7.* with noncanonical splice junction removal and output of unmapped reads. Coordinate sorting of BAM files using samtools v1.21. UMI based de‐duplication: Removal of PCR and optical duplicates using umicollapse v1.1.0. Mapping QC: Alignment quality metrics, strand specificity, and read distribution across genomic features using rustqc v0.2.1. Generation of comprehensive QC report using multiqc v1.33. Gene expression quantification using featureCounts (subread package v2.1.1) with strand‐specific counting, multi‐mapping read fractional assignment, exons and three prime UTR as the feature identifiers, and grouped by gene_id. Final gene counts were annotated with gene biotype and other metadata extracted from the reference GTF file. Functional enrichment performed using gene set enrichment analysis with gseapy v0.12 using the MSigDB Hallmark gene set.

### 2.9 Flow cytometry

Following 3 days of HD treatment, HCC‐1428 and HCC‐1428/LTED cells were seeded in triplicate in a 12‐well plate at 150 000 cells per well. For BrdU labeling studies, cells were treated as indicated ±1 nm E2 and 150 nm or 500 nm entinostat for 24 h or 72 h in HD medium. Two to three hours prior to cell harvest, 10 μm BrdU was spiked into the medium. Cells were trypsinized and immunostained for BrdU, phosphorylated H2AX‐S139 (γH2AX), cleaved PARP, and DAPI using the BD Pharmingen apoptosis, DNA damage, and cell proliferation kit (BD Biosciences, Franklin Lakes, NJ, USA, #562253, RRID: AB_2869407). Cells were analyzed on a BD FACSCelsta Multicolor flow cytometer, and results were analyzed using flowjo software. For apoptosis studies, HD cells were pretreated ±300 nm entinostat for 3 days, followed by treatment ±1 nm E2 (± continued entinostat) for 7 days; cells were harvested and analyzed on Day 10; medium and drugs were changed 3 days prior to harvest to ensure 3 days' worth of dead cells would be collected. Apoptosis staining was performed using TACS Annexin V‐FITC Apoptosis Detection Kit (R&D Systems #4830‐250‐K). Samples were analyzed on a MACSQuant, and data were analyzed using flowjo.

### 2.10 Immunofluorescence

#### 2.10.1 Cell lines

Cells were HD for 3 days, reseeded onto glass coverslips, allowed to adhere overnight, and then treated ±1 nm E2 and 500 nm entinostat for 24 h. Cells were fixed with 1.85% formaldehyde and permeabilized by incubation in 0.1% Triton X‐100 in PBS for 10 min. Cells were washed with TBS‐T and blocked with 1% BSA in TBS‐T for 1 h at room temp. Primary antibody (phospho‐histone H2AX_S139_, γH2AX, Cell Signaling Technology #9718) was diluted 1 : 300 in blocking buffer and incubated with cells on a slow rocker at 4 °C overnight. Cells were washed with TBS‐T, and primary antibody was detected by incubation in the dark with fluorophore‐labeled anti‐rabbit Alexa488‐conjugated secondary antibody (Thermo Scientific #A11008) for 1 h at room temp. Cells were washed in TBS‐T and PBS, and coverslips were mounted on slides with ProLong Gold with DAPI mounting medium (Thermo Scientific #P36931). Cells were imaged using Leica SP8 Upright and Zeiss LSM 800 confocal microscopes. γH2AX foci per nucleus were quantified using CellProfiler software (RRID: SCR_007358) in ≥ 100 nuclei per condition.

#### 2.10.2 Tumors

FFPE tissues were cut into 5‐micron sections mounted on slides, deparaffinized in xylene, and rehydrated in a graded ethanol sequence. Antigen retrieval was performed in citrate buffer, pH 6 (Sigma‐Aldrich #C999) in a decloaking chamber (Instapot Pressure Cooker) on high pressure for 20 min. All washes, blocking, and staining were performed in a humidity chamber. Tissues were blocked in 5% BSA with 0.3% Triton X‐100 in PBS for 1 h, and washed in 3% BSA in PBS. The Click‐iT EdU cell proliferation kit for imaging (Thermo Scientific #C10337) was utilized to perform an azide reaction to detect EdU. Primary antibody (anti‐mouse phospho‐histone H2AX_S139_, γH2AX, Cell Signaling Technology #80312; anti‐rabbit cleaved caspase 3, Cell Signaling Technology #9664) was diluted in 1% BSA with 0.3% Triton X‐100 in PBS and incubated at 4 °C overnight. Tissue sections were washed with PBS and incubated with anti‐rabbit Alexa594 or anti‐mouse Alexa647 secondary antibodies (Thermo Scientific #A11012 and #A21235) with 1% BSA and 0.3% Triton X‐100 in PBS for 1.5 h at room temp. Slides were washed with PBS, treated with TrueVIEW Autofluorescence Quenching Kit (Thermo Scientific #SP‐8400‐15), and mounted in ProLong Glass Antifade mounting medium with Vector NucBlue Stain (Thermo Scientific #P36985). Slides were allowed to cure overnight and imaged on a Leica SP8 Upright Confocal Microscope. Fluorescent signal was quantified using cellprofiler software.

### 2.11 Statistical analysis

Cell growth data and immunofluorescence scores were analyzed by *t*‐test (for 2‐group experiments) or ANOVA followed by Bonferroni multiple comparison‐adjusted *post hoc* testing between groups (for experiments with ≥ 3 groups). Tumor volumes were analyzed using linear mixed modeling: log_10_ (tumor volume_*it*_) = *a*_*i*_ + *b* * *t* + *e*_*it*_, where *i* represents the *i* ^th^ mouse, *t* represents the time to tumor volume measurement, *a*_*i*_ represents the mouse‐specific log tumor volume at t = 0, b represents the rate of tumor volume growth, and *e*_*it*_ represents deviation of measurements from the model over time [31, 32]. Mouse heterogeneity (baseline tumor volume) is denoted by variance of *a*_*i*_, and *b* * log_e_(10) * 100 indicated tumor volume increase percentage per week. Treatment groups were assessed using a z‐test for the slopes with standard error derived from the output of the function lme from library nlme in r. Based on the concept of cancer cell surviving fraction [33], we used tumor volume data before tumors became nonpalpable due to drug treatment to determine short‐term synergy based on log_10_ (surviving tumor cell fraction) as described [34]. Tumor recurrence curves were compared using log‐rank test.

## 3 Results

### 3.1 HDACi increases histone acetylation and chromatin accessibility

Chromatin organization is determined in part by covalent marks applied to DNA and histone tails, which affects transcription. HDACs function as ‘erasers’ of acetylation marks on histone tails, facilitating chromatin compaction and preventing transcription [35], and treatment with an HDACi blocks removal of acetylation. We evaluated chromatin organization in two ER^+^ BC cell line models that are growth‐inhibited by E2 (HCC‐1428/LTED cells and MDA‐MB‐415/ESR1 cells that overexpress endogenous and exogenous ER and exhibit hormone‐independent growth [17]) and their isogenic counterparts (HCC‐1428 and MDA‐MB‐415/Luc). Acetylation of histone H3 on lysine‐27 (H3K27ac) increased in a dose‐dependent manner after 24 h of treatment with the HDAC1/3‐selective inhibitor entinostat in ER^+^ BC cells (Fig. 1A). Treatment with another class I‐selective HDACi, mocetinostat, also induced H3K27ac (Fig. S1A).

**Fig. 1:**
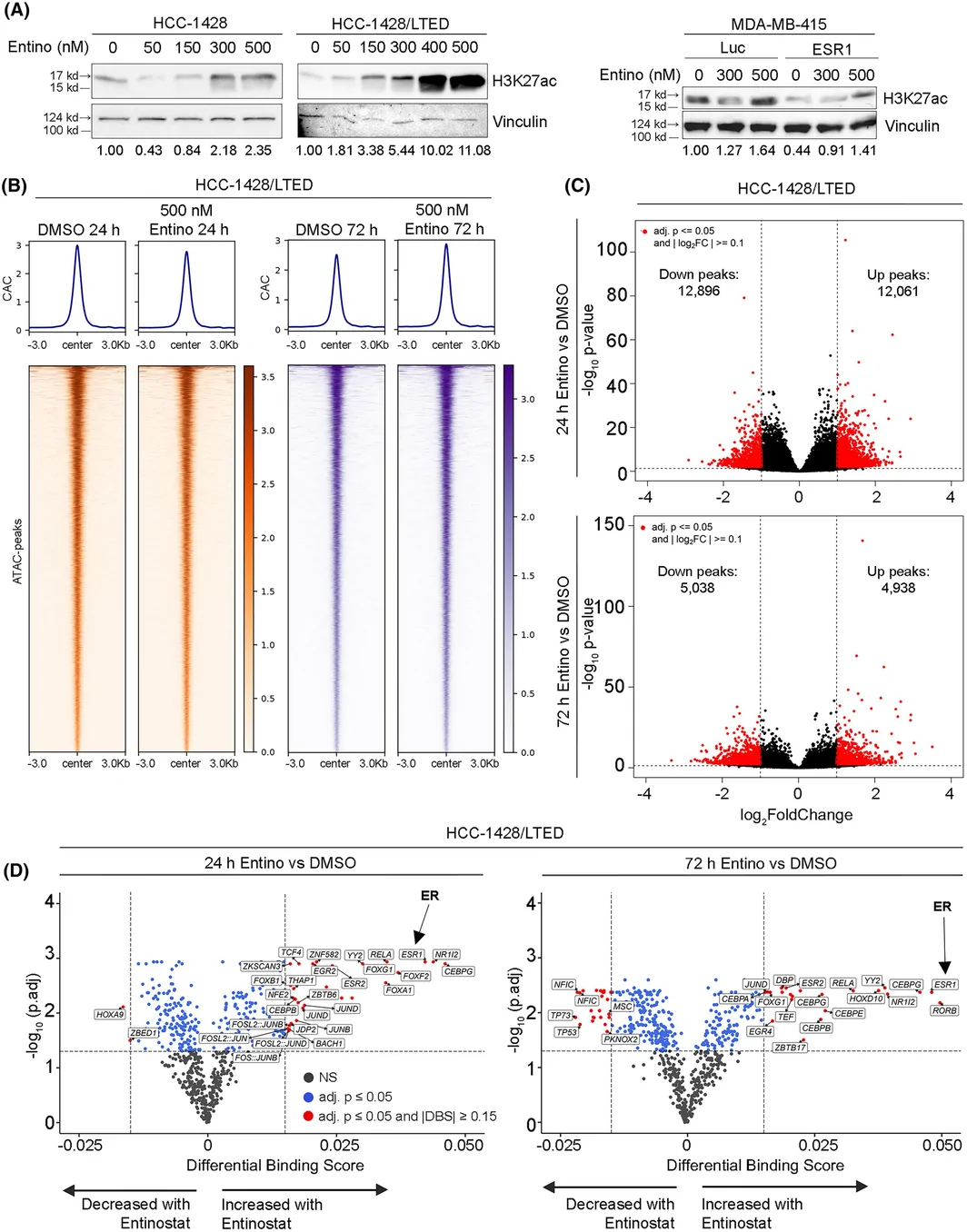
HDACi increases histone acetylation and chromatin accessibility. (A) Cells were treated with entinostat at the indicated concentrations for 24 h, and lysates were analyzed by immunoblot. Relative band intensity is noted below each lane. Results shown are representative of 3 independent experiments. (B) Cells were treated in triplicate ±500 nm entinostat for 24 or 72 h and processed for ATAC‐seq. Accessibility of genomic features ±3 kb from the peak center is displayed in tornado plots. Tn5 insertion frequency is shown in signal plot at top. CAC: chromatin accessibility coverage. (C) Differential accessibility analysis of ATAC‐seq data. Data points with adjusted *P* ≤ 0.05 and |log_2_(fold‐change)| ≥ 1 are colored red. (D) Transcription factor motif enrichment analysis of ATAC‐seq peaks. Red color indicates a significant differential binding score (DBS) and significant adjusted *P*‐value with entinostat treatment compared to vehicle.

To assess the effect of HDACi, we measured chromatin accessibility with the assay for transposase‐accessible chromatin sequencing (ATAC‐seq). Tornado plots centered around the most accessible chromatin regions at baseline (~ 10 000 peaks), show the average accessibility profile across dominant open chromatin sites in HCC‐1428/LTED cells treated with entinostat for 24 or 72 h. (Fig. 1B). Differential chromatin accessibility was tested using all peaks genome‐wide, which revealed both increased and decreased accessibility after 24 and 72 h of entinostat (Fig. 1C); this suggests that short‐term entinostat induced chromatin remodeling at specific regulatory elements rather than widespread changes. To examine whether HDACi altered chromatin accessibility in regions associated with transcriptional programs, we conducted motif enrichment analysis. Entinostat primarily elicited positive differential binding scores for transcription factor motifs including that of *ESR1* (ER) at both time points (Fig. 1D), suggesting entinostat increases accessibility of genomic regions involved in ER signaling in hormone‐depleted (HD) conditions.

### 3.2 Combination therapy with entinostat and 17b‐estradiol inhibits cell growth and induces apoptosis

HCC‐1428 cells are growth‐induced by E2. The addition of entinostat in the E2‐supplemented setting resulted in a significant growth disadvantage (Fig. 2A). By contrast, HCC‐1428/LTED cells thrive in HD conditions and are growth‐inhibited by E2 [17]. The addition of entinostat caused a dose‐dependent growth disadvantage to HCC‐1428/LTED cells in HD conditions, and the combination of entinostat/E2 was more effective than single agents (Fig. 2A). In MDA‐MB‐415/Luc control cells, growth was unaffected by E2, but cells were growth‐inhibited by entinostat. MDA‐MB‐415/ESR1 cells grow in HD conditions and respond to entinostat. These cells are growth‐inhibited by 10 nm E2, and combined E2/entinostat induced near‐complete growth inhibition (Fig. 2A). Similarly, the HDACi mocetinostat inhibited and enhanced the growth‐suppressive effect of E2 (Fig. S1B). We also tested if the growth‐suppressive effects of E2/HDACi combination therapy are partly due to apoptosis. Prior work showed E2 increases apoptosis after ~ 6 days of treatment [18]. Since E2 rapidly induces ER transcriptional signaling and yields DNA damage within 24 h [18], we primed HCC‐1428/LTED cells by treatment ± entinostat for 3 days followed by run‐in treatment ± E2 for 7 days. E2 and entinostat each induced apoptosis, and the combination was most effective (Fig. 2B).

**Fig. 2:**
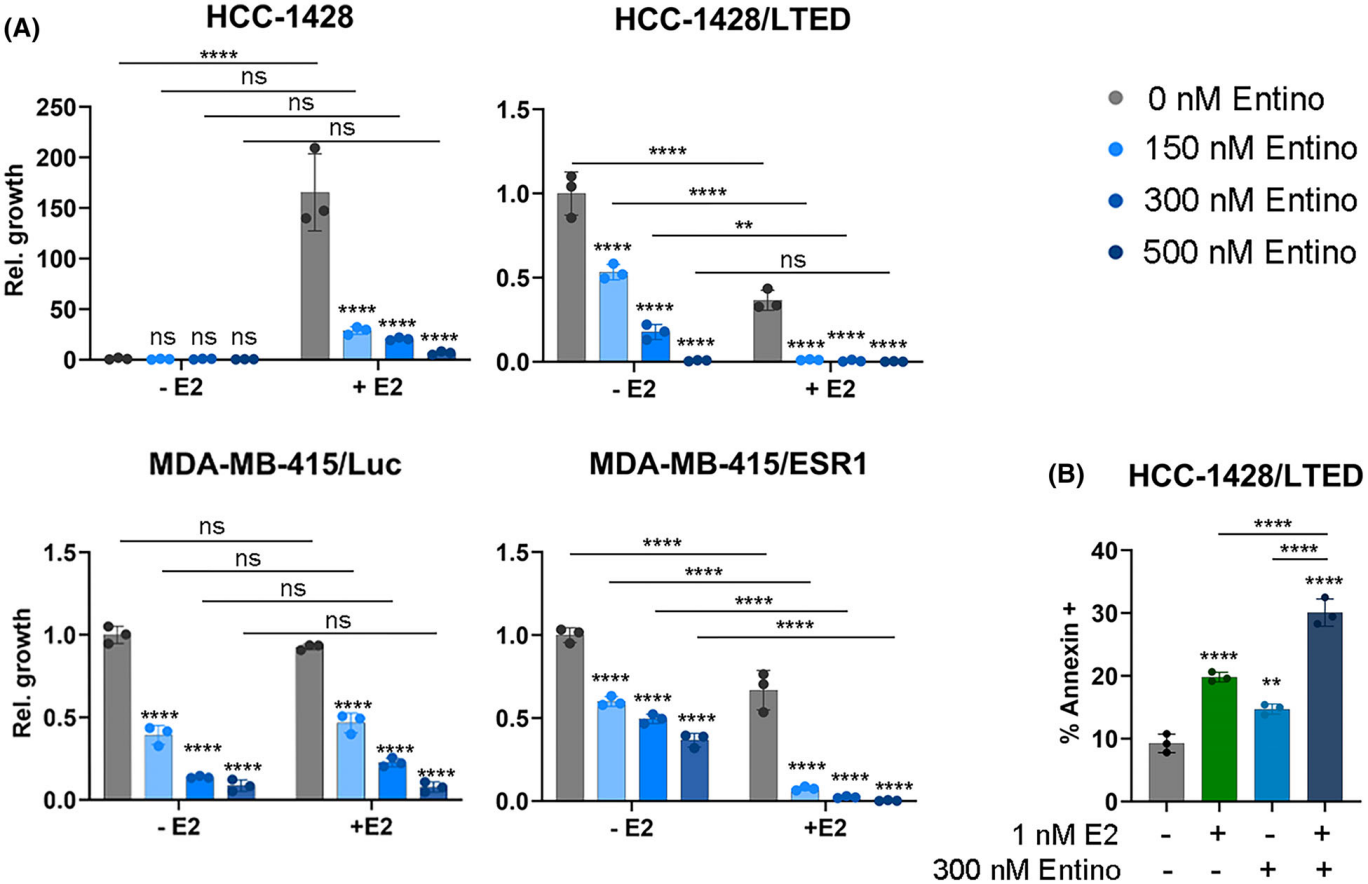
HDACi and E2 combine to inhibit cell growth and induce apoptosis. (A) Cells were seeded in triplicate in HD medium ± E2 ± entinostat for 28 days. HCC‐1428/LTED cells were treated ±1 nm E2 and MDA‐MB‐415 cells were treated ±10 nm E2. (B) Cells in HD medium were pretreated in triplicate ±300 nm entinostat for 3 days followed by the addition of E2 for 7 days. Medium/drugs were changed 3 days prior to endpoint when cells were analyzed for apoptosis by annexin V labeling and flow cytometry. Bars represent mean ± SD. Results shown are representative of 3 independent experiments. **P ≤ 0.01, ****P ≤ 0.0001 by Bonferroni‐adjusted *post hoc* test compared to each respective ‘0 nm Entino’ group (A) or control (B) unless otherwise indicated. ns, not significant.

### 3.3 Entinostat and E2 drive distinct gene expression profiles

ER is a transcription factor canonically activated by estrogens and binds estrogen response element (ERE) motifs in DNA. Prior evidence suggested that E2 therapy acts in part via induction of a transcriptional phenotype that induces DNA damage [18]. Based on our observations that entinostat increased chromatin accessibility and suppressed growth in combination with E2 in endocrine‐resistant cells, we tested the potential effects on genes whose expression is canonically induced by E2: *TFF1*, *PDZK1*, and *AREG* [36]. RT‐qPCR analysis showed entinostat variably and temporally affected hormone‐independent and E2‐induced gene expression across model systems (Fig. S2A–D), prompting a more comprehensive genome‐wide analysis.

HCC‐1428 and HCC‐1428/LTED cells were HD for 3 days and treated ± entinostat for 1, 3, or 7 days to evaluate the short‐ and long‐term effects of HDACi. Cells were treated ± E2 for the final day prior to RNA harvest to enable evaluation of the temporal effects of HDACi without confounding effects from different durations of E2 exposure (Fig. 3A). Principal components analysis (PCA) showed most of the transcriptomic variance was due to adaptation to hormone‐independent growth. Transcriptomic profiles of entinostat‐treated cells were distinct from those of nonentinostat‐treated cells, suggesting entinostat had a substantial influence (Fig. S3). The 500 most variably expressed genes (with largest coefficients of variation) within each cell line are displayed in heatmaps for visualization (Fig. 3B,C). Overlap analysis revealed HCC‐1428/LTED cells had fewer genes altered by E2 compared to parental cells, and combination E2/entinostat induced unique gene expression changes not elicited by single‐agent treatments (Fig. S4A,B). While E2 promoted up‐ and down‐regulation of genes, entinostat primarily induced transcriptional upregulation, consistent with the notion that increased histone acetylation increases chromatin accessibility permissible to transcription. Cotreatment with E2 plus entinostat for 1 days maintained the skewed distribution of differentially expressed genes in favor of upregulation (Fig. 3D); this phenomenon was consistent among samples treated for 3 or 7 days with entinostat ±1 day of E2 (Fig. S5A,B).

**Fig. 3:**
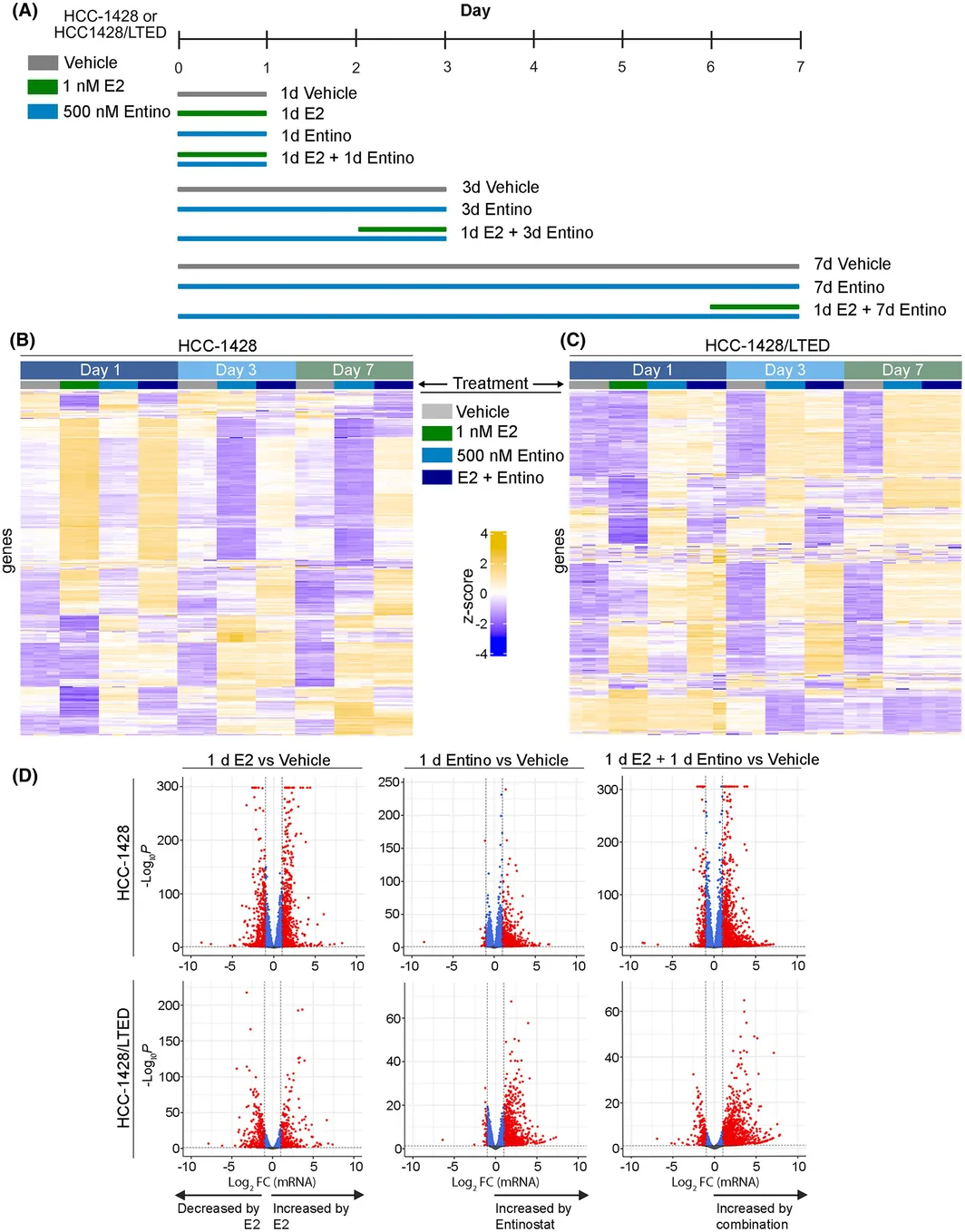
Transcriptomic analysis reveals temporal responses to E2 and HDACi. RNA‐seq was performed using HCC‐1428 and HCC‐1428/LTED cells in HD medium treated in triplicate as described in (A). (B, C) Heatmap of the 500 most variably expressed genes across all treatment groups within each cell line. (D) Volcano plots of differentially expressed genes after 1 day of drug treatments. Red dots indicate significantly altered genes (*P* ≤ 0.05) with |log_2_ fold‐change| ≥ 1.

Gene set enrichment analysis (GSEA) showed E2‐induced transcriptomic changes reflecting ‘estrogen response’, ‘MYC targets’, and ‘E2F targets’ (which are downstream of ER signaling [37]) in both parental and LTED cells (Fig. 4). Entinostat elicited opposite effects in both cell lines, inducing negative enrichment for ‘E2F targets’, ‘MYC targets’, and ‘DNA repair’ gene sets. Entinostat also induced positive enrichment for ‘estrogen response’ gene sets in HCC‐1428/LTED cells but not HCC‐1428 cells, suggesting that the estrogen‐independent growth phenotype of LTED cells may shift response to HDACi; these findings concur with data in Fig. 1D showing entinostat induced opening of chromatin enriched for ER binding motifs. Notably, parental and LTED cells showed different transcriptomic responses to the combination of E2/entinostat. In HCC‐1428 cells, overlapping gene sets were positively enriched in response to E2 or E2/entinostat, but negatively enriched by entinostat (Fig. 4A). In contrast, LTED cells showed overlapping gene sets enriched by entinostat or E2/entinostat (Fig. 4B). These findings suggest that E2 elicited a dominant transcriptional effect in parental cells, and entinostat elicited a dominant effect in LTED cells.

**Fig. 4:**
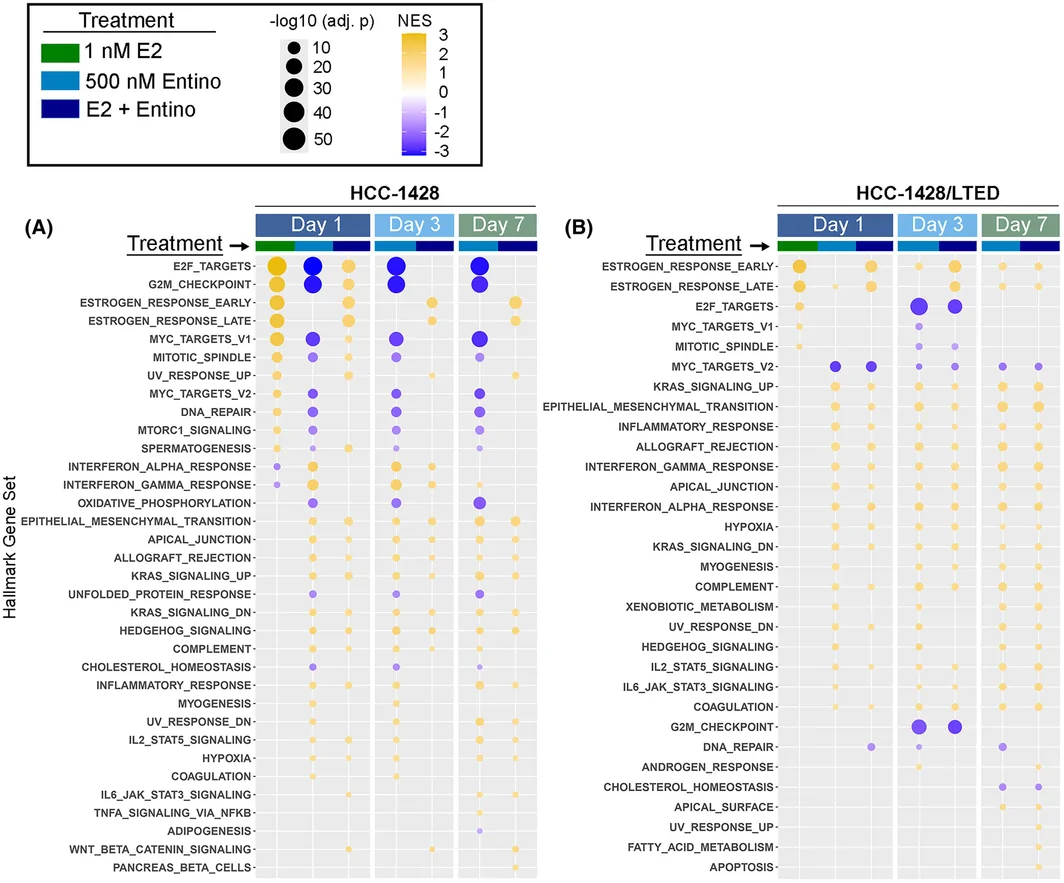
E2 and HDACi impact cell cycle, DNA repair, and estrogen response pathways in HCC‐1428 and HCC‐1428/LTED cells. Gene set enrichment analysis of transcriptomic data (from Fig. 3) using Hallmark pathways. mRNA expression compared to vehicle control at each time point was used for each drug treatment group in HCC‐1428 (A) and HCC‐1428/LTED (B) cells. Pathways were ranked vertically by enrichment in E2‐treated cells. Day 1, 3, or 7 refers to the duration of entinostat treatment as in Fig. 3A. NES, normalized enrichment score.

### 3.4 Entinostat alters the E2‐induced DNA binding profile of ER

To understand how ER binding was altered by drug treatments in endocrine‐resistant cells, and how this associated with transcriptional changes, we performed CUT&RUN to map ER binding sites (peaks) across the genome in HCC‐1428/LTED cells pretreated for 3 days ± entinostat, and then treated ± E2 for 1.5 h. Peak analysis showed ER primarily bound intergenic and promoter regions in response to treatment with E2 ± entinostat (Fig. 5A and Table S2). Differential peak analysis showed E2 generally increased ER binding while entinostat reduced binding (Fig. 5B). Single‐agent E2 induced the most ER binding sites (1616 peaks not detected in vehicle‐treated cells), 1262 of which were unique to E2 treatment. Entinostat cotreatment decreased the number of E2‐induced ER binding sites (Fig. 5C). Single‐agent entinostat induced only 7 peaks that were not detected in vehicle‐treated cells. ER‐bound genomic regions induced by E2 were significantly enriched for estrogen response element (ERE) motifs regardless of cotreatment ± HDACi (Table S3). The combination of E2/entinostat induced 359 peaks not found in vehicle‐treated cells, of which 344 (95.8%) overlapped with those induced by E2 but not entinostat. We identified the genes associated with those 344 peaks, and leveraged transcriptomic data (Fig. 3) from LTED cells treated ± entinostat for 3 days ± E2 for the final day. Transcriptional profiles induced by E2 were partly altered by entinostat cotreatment (Fig. 5D), suggesting that HDACi changes the E2‐regulated transcription of genes with proximal ER binding sites. Hallmark gene set enrichment analysis of clusters of genes revealed that entinostat, E2, and the combination each induced expression of transcripts enriched for ‘estrogen response’, but distinct sets of genes supported such enrichment (Fig. 5D).

**Fig. 5:**
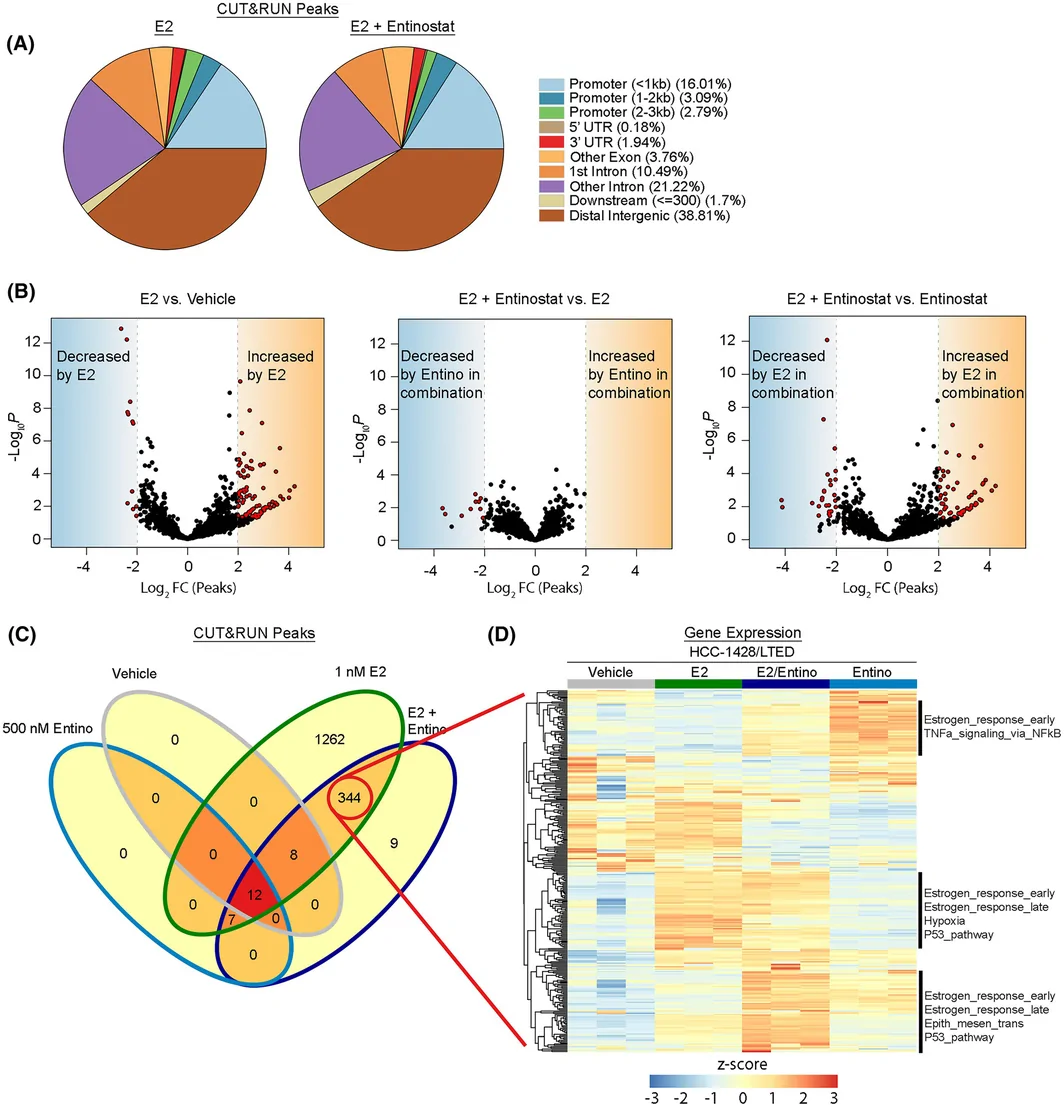
E2 induces ER binding that is altered by HDACi. HCC‐1428/LTED cells were treated in duplicate ±500 nm entinostat for 3 days and ± 1 nm E2 for the final 1.5 h. Samples were processed for CUT&RUN with antibodies targeting ER. (A) Peak distribution of ER binding sites in promoters, 5′ UTR, 3′ UTR, introns, or exons. (B) Volcano plots showing differential binding of ER with a log_2_FC ≥ 2 across treatments. (C) Venn diagram of peaks across drug treatments. (D) Heatmap of gene expression levels of genes proximal to the 344 overlapping peaks from E2 and E2/entinostat groups in (C). mRNA data were derived from cells treated ± entinostat for 3 days ± E2 for the final day as in Fig. 3A.

### 3.5 Combination therapy induces DNA damage, and HDACi halts cell cycle progression

GSEA of transcriptomic data showed negative enrichment for the ‘G2M checkpoint’ and ‘DNA repair’ gene sets in entinostat‐ and combination‐treated LTED cells (Fig. 4B). To investigate the functionality of these inferences, we simultaneously assayed DNA replication (BrdU incorporation), cell cycle stage (DAPI), and DNA damage (γH2AX). E2 increased the proportion of HCC‐1428 cells in S‐phase that was prevented by entinostat pretreatment (Fig. 6A). In contrast, entinostat pretreatment decreased the S‐phase fraction of HCC‐1428/LTED cells that was partly rescued by E2; both conditions increased the proportion of G1 cells relative to vehicle (Fig. 6A). These observations were reinforced when measuring proportions of replicating cells: E2 induced replication in both cell lines; entinostat pretreatment suppressed replication that was partly rescued by E2 (Fig. 6B). Combination treatment induced the greatest amount of DNA damage in both cell lines (Fig. 6C). A lower dose of entinostat (150 nm; Fig. S6A–C) was insufficient to elicit the phenotypes observed with 500 nm entinostat (Fig. 6). Although entinostat‐treated cells showed limited replication, replicating cells showed the most DNA damage (Fig. 6D and Fig. S7). Inhibition of replication/cycling by single‐agent entinostat, rescue of replication/cycling and transcriptional modulation by E2 cotreatment, and increased DNA damage in cycling cells suggest competing mechanisms of cell cycle progression and inhibition underlie the anticancer efficacy of combination therapy.

**Fig. 6:**
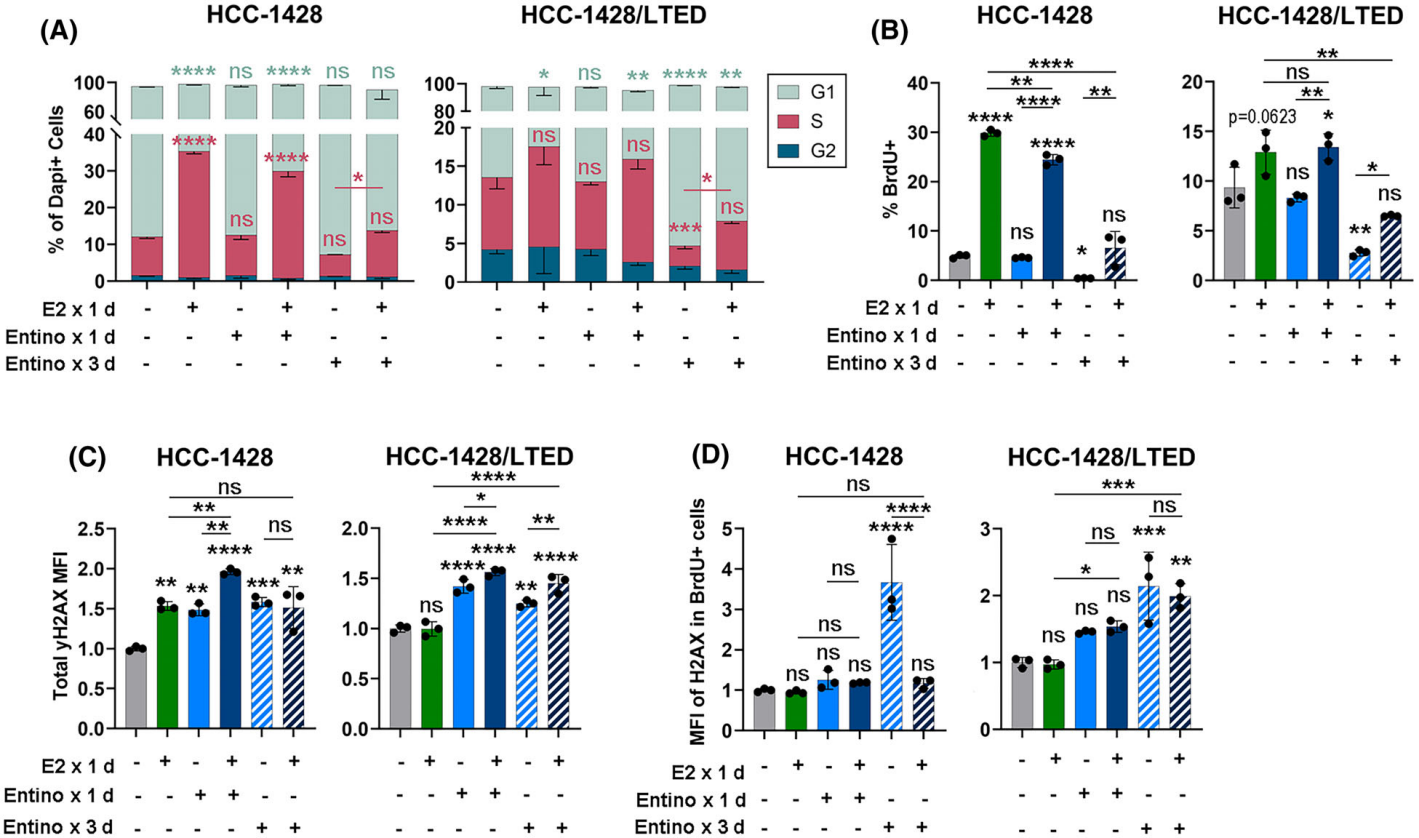
HDACi slows cell cycling, and the addition of E2 induces DNA damage in replicating cells. HCC‐1428 and HCC‐1428/LTED cells were treated with HD medium ±1 nm E2 ± 500 nm entinostat as indicated in triplicate. BrdU was spiked into media 3 h prior to cell harvest. Cells were immunostained for BrdU, cleaved PARP, and γH2AX with DAPI counterstain and analyzed by flow cytometry. (A) Proportions of cells in phases of the cell cycle as measured by DAPI signal. (B) Proportions of replicating cells as measured by BrdU positivity. (C, D) DNA damage as measured by γH2AX signal in all cells (C) and in replicating cells (D). Cells staining positively for cleaved PARP were excluded from γH2AX analysis. Data are presented as triplicate populations of ≥ 10 000 cells, mean fluorescence intensity (MFI) ± SD. *P ≤ 0.05, **P ≤ 0.01, ***P ≤ 0.001, ****P ≤ 0.0001 by Bonferroni‐adjusted *post hoc* test compared to respective control group unless otherwise indicated. ns: not significant.

### 3.6 E2/entinostat combination therapy is synergistic *in vivo*

Two estrogen‐independent PDX models of ER^+^ BC, WHIM16 and CTG‐3346, which are, respectively, *ESR1*‐amplified and *ESR1*‐nonamplified, were grown in ovariectomized NSG mice [15, 18]. Mice bearing tumors ~ 200 mm^3^ were randomized to treatment: vehicle; E2 (via s.c. pellet); entinostat (10 mg·kg^−1^·day^−1^ p.o. given 5 days·week^−1^ for 7.5 weeks); E2/entinostat. In both models, entinostat modestly slowed tumor growth compared to vehicle (Fig. 7A and Fig. S8A,B). E2 and combined E2/entinostat induced complete regression in all cases; following cessation of entinostat after 7.5 weeks, mice were monitored for tumor recurrence (defined as regrowth to baseline volume). Mice treated with single‐agent E2 showed shorter time to recurrence than combination‐treated mice (Fig. 7B). Entinostat provided synergy with E2 to reduce the surviving fraction of tumor cells in CTG‐3346 tumors (*P* = 0.0012) and WHIM16 tumors (*P* = 0.0537). Mice bearing fully regressed WHIM16 tumors were rerandomized to second‐line treatment with E2 ± mocetinostat. Combined E2/mocetinostat slowed tumor growth compared to single‐agent E2 (Fig. S9), suggesting (a) tumors that recurred during E2 treatment (± prior entinostat cotreatment) remained sensitive to combined E2/HDACi therapy, and (b) longer‐term first‐line combination treatment may have provided more durable protection against recurrence.

**Fig. 7:**
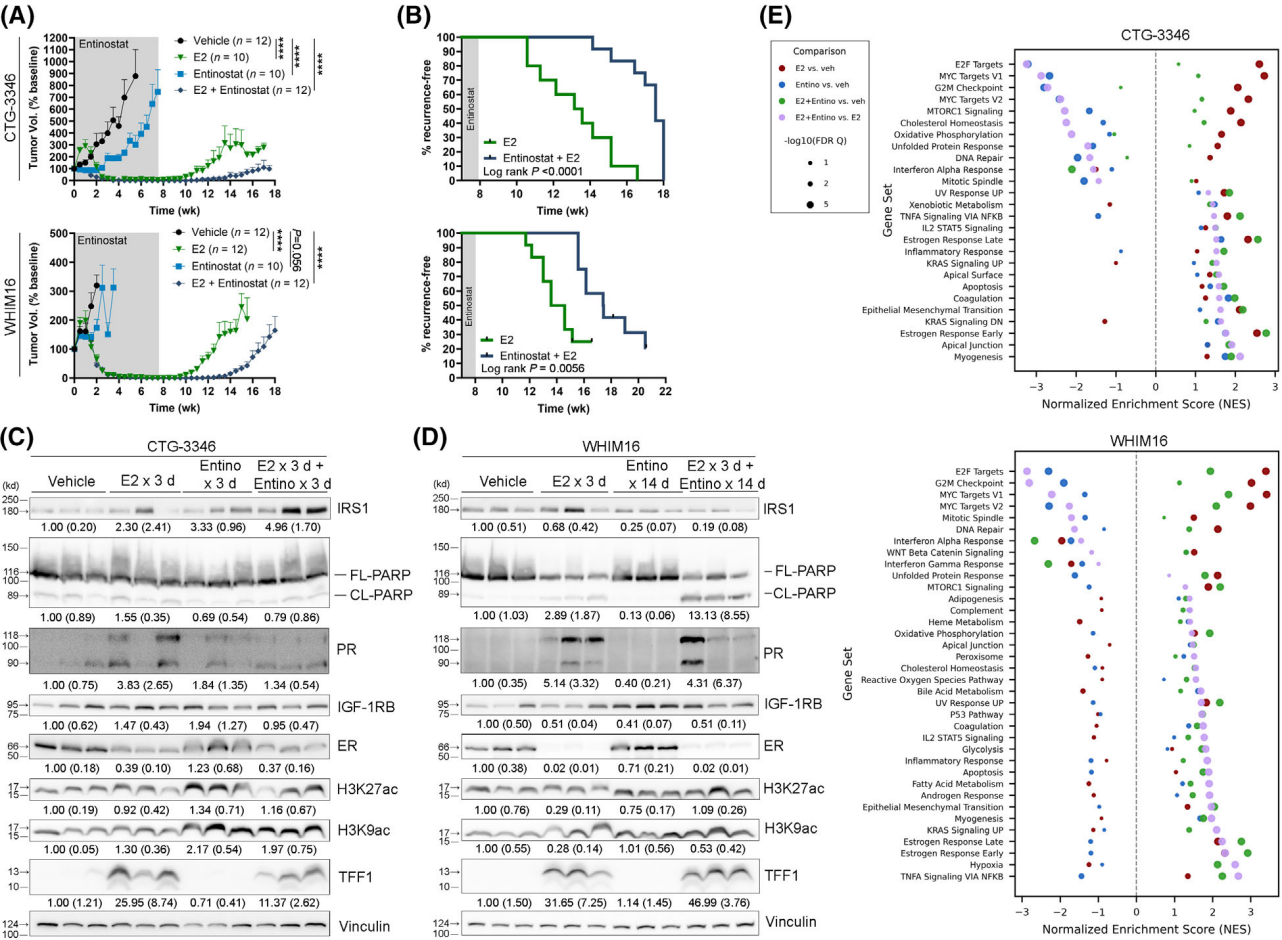
E2 and HDACi synergistically induce tumor regression and prevent recurrence. (A, B) Ovx mice bearing CTG‐3346 or WHIM16 tumors were randomized to drug treatments; animal numbers are indicated in keys. E2 was delivered continuously, and entinostat was administered 5 days·week^−1^ for 7.5 weeks (gray shading). Tumor growth curves (A) are shown as mean + SEM. ****P < 0.001 by linear mixed modeling of raw tumor volumes. After 7.5 weeks of E2 ± entinostat treatment, mice with completely regressed tumors were monitored for recurrence in (B), defined as regrowth to 200 mm^3^. (C, D) Tumors were harvested 4 h after drug treatment on Day 3 or 14. Tumor lysates (*n* = 3/group) were analyzed by immunoblot. Mean (SD) band intensities for each treatment group are noted below each blot. Cleaved PARP was normalized to full‐length PARP prior to normalization to vinculin loading control. (E) Tumors from (C/D) were analyzed by RNA‐seq. Transcriptomic profiles were analyzed by GSEA using Hallmark pathways. Dot color reflects NES for comparison of each treatment group vs. vehicle, or the drug combination vs. E2 as indicated.

Immunoblot analysis of tumors confirmed entinostat inhibited HDACs, as reflected by increased levels of histone 3 Lys acetylation (Fig. 7C,D). E2 increased levels of TFF1, which is encoded by an ER‐inducible gene, and decreased ER levels through a canonical negative feedback loop. E2 variably induced the expression of IRS‐1, IGF‐1Rb, or PR, which are also encoded by ER‐inducible genes. E2/entinostat induced more apoptosis than single‐agent treatments in CTG‐3346 tumors as reflected by increased PARP cleavage. Entinostat induced ER accumulation (Fig. 7C,D) driven in part by its transcriptional upregulation; however, drug treatments elicited variable effects on canonical ER‐inducible transcripts. WHIM16 tumors are *ESR1*‐amplified and highly sensitive to E2, as shown by the stark upregulation of *AREG, PDZK1*, and *TFF1* by E2 (Fig. S10A,B).

Transcriptomic profiling of tumors revealed E2 induced significant enrichment for gene sets reflective of ‘estrogen response’, ‘MYC targets’, and ‘E2F targets’ (Fig. 7E), consistent with effects observed *in vitro* (Fig. 4). Entinostat had opposing effects on pathways downstream of ER, including ‘MYC targets’ and ‘E2F targets’, but entinostat increased enrichment for ‘estrogen response’ pathways in tumors; these patterns are consistent with results from cultured HCC‐1428/LTED but not parental HCC‐1428 cells, suggesting overlapping transcriptomic phenotypes among endocrine‐resistant models. Also aligned with LTED cell results (Fig. 4), the combination of E2 plus entinostat induced gene set enrichment profiles often akin to that of single‐agent entinostat (Fig. 7E), suggesting entinostat has a dominant effect on select pathways in these endocrine‐resistant tumors.

Mice bearing CTG‐3346 tumors were injected with EdU 3 h prior to tumor harvest to label replicating cells. E2 increased proportions of replicating tumor cells, which was suppressed by entinostat (Fig. S11A,B), as was observed *in vitro* (Fig. 6B). Replicating (EdU^+^) tumor cells showed increased DNA damage compared to nonreplicating cells. E2 induced DNA damage (γH2AX) that was suppressed by entinostat (Fig. S11C).

## 4 Discussion

We modeled endocrine‐resistant ER^+^ BC with long‐term estrogen‐deprived (LTED) cells, genetically engineered ER^+^ BC cells that overexpress ER, and patient‐derived xenograft (PDX) models that grow independently of estrogen. While E2 and entinostat were each moderately effective at preventing growth of endocrine‐resistant cells, these agents were more effective in combination. Entinostat and E2 promoted opposing cell cycling mechanisms. Transcriptomic and CUT&RUN analyses showed that entinostat modulated the response to E2, suggesting an effect of HDACi on the ER signaling axis. Finally, combination treatment was synergistically effective at inducing tumor regression and delaying time to recurrence in PDX models.

HDACi targets tumor cells through mechanisms including, but not limited to, cell cycle arrest, cell death induction, differentiation, immune modulation, upregulation of oxidative stress, impaired DNA damage repair, and angiogenesis reduction [38, 39, 40, 41, 42]. Cancer cells have been found to exhibit ≥ 10‐fold sensitivity to HDACi compared to noncancer cells *in vitro* [39]. It is thought that cancer cells are more vulnerable to class I (HDAC1/2/3/8)‐selective HDACi due to reliance on class I HDAC enzymes for epigenetic regulation while noncancer cells maintain redundant mechanisms of epigenetic regulation. Noncancer cells that have redundant HDAC enzymes may be vulnerable to pan‐HDACi, which would account for the success—via increased therapeutic index—of selective HDACi as cancer therapeutics [43].

Class I HDACs play essential roles in chromatin remodeling to facilitate DNA damage repair. HDAC1/2 are recruited to sites of double‐stranded DNA breaks to deacetylate histones 3/4, enabling DNA repair through nonhomologous end joining [38]. Beyond histone acetylation, HDACs regulate the ATM pathway to modulate the DNA damage response [44]. Thymidylate synthetase and CTP synthase are involved in DNA synthesis and repressed by HDACi, which could contribute to cell cycle arrest [45]. As supported by our results showing entinostat induces DNA damage and cell death (Figs 2B, 4 and 6C,D), pharmacological inhibition of HDACs disrupts DNA damage repair processes and increases vulnerability to DNA damage‐induced cell death. Additionally, ER and HDACi each enhance the formation of DNA–RNA hybrids called R‐loops implicated in the therapeutic action of E2 [18, 46], which may be a point of mechanistic convergence of estrogen and HDACi therapies.

Accumulation of histone acetylation is a widely used marker to reflect HDAC inhibition that is canonically associated with chromatin opening [47]. Colorectal cancer patient‐derived organoids treated with the HDACi SAHA for 6 h showed a gain in accessible peaks via ATAC‐seq [48]. Cutaneous T‐cell lymphoma cells in patients that clinically responded to the HDACi romidepsin or vorinostat showed concurrent increases in chromatin accessibility over the course of 4 weeks, while patients who had marginal responses saw decreases; this offers chromatin accessibility as a potential biomarker of HDACi response [19]. Entinostat increased histone 3 acetylation within 24 h in ER^+^ BC cells, and chromatin accessibility progressively changed up to the final evaluated time point of 72 h (Fig. 1A–C). Although HDACi is expected to increase chromatin accessibility, we detected both increases and decreases in accessibility with entinostat treatment of endocrine‐resistant ER^+^ BC cells (Fig. 1C). Examples of HDACi‐induced co‐occurring gains and reductions in chromatin accessibility have also been reported in other solid tumor cell lines [48, 49, 50]. Transcriptional changes induced by entinostat were apparent within 1 day, but some genes took longer (3–7 days) to show alterations (Fig. 3B), potentially due to secondary/downstream effects. We therefore must consider whether such downstream transcriptional effects of entinostat shaped chromatin accessibility. Given that entinostat increased accessibility of DNA regions enriched for several transcription factor motifs including ER in ER^+^ BC cells, but few motifs were identified in DNA regions that were closed upon HDACi (Fig. 1D), it is also possible that the closed regions were not proximal to active genes in this system.

The transcriptional changes induced by entinostat (with or without E2) were largely in the direction of increased gene expression (Fig. 3D and Fig. S5). Despite the entinostat‐induced enrichment for ER binding motifs in the newly opened chromatin (Fig. 1D), entinostat decreased the number of loci bound by ER upon short‐term stimulation with E2 (Fig. 5C); whether longer‐term E2 treatment, which induces the expression of ER cofactors [51], would have enabled ER binding at more loci is a subject for future investigation. Among the 344 ER‐bound loci induced by E2 regardless of the presence of entinostat, entinostat cotreatment altered the expression of a substantial number of genes compared to single‐agent E2 (Fig. 5D). Entinostat elicited negative enrichment for gene sets associated with E2F_Targets and G2M_Checkpoint pathways in both HCC‐1428 and HCC‐1428/LTED cells; in contrast, E2 elicited positive enrichment for these gene sets. Combined E2/entinostat caused positive enrichment for those gene sets in parental cells, but negative enrichment in LTED cells (Fig. 3), suggesting that E2 has a dominant transcriptional effect in (endocrine‐sensitive) parental cells while HDACi has a dominant effect in (endocrine‐resistant) LTED cells.

Comparison of our findings with prior reports of mechanisms underlying therapeutic response to estrogen suggests there are (a) multiple pathways of response and/or (b) multiple downstream mechanisms differentially engaged. The majority of preclinical studies of estrogen therapy used endocrine‐resistant derivatives of MCF‐7 ER^+^ BC cells and reported therapeutic mechanisms including loss of glutathione synthesis, ER‐induced upregulation of apoptotic genes, inflammation, translational load, endoplasmic reticulum stress (unfolded protein response), and ER‐induced up‐ or down‐regulation of NF‐κB signaling [16, 52, 53, 54, 55, 56, 57, 58, 59]. We found that estrogen therapy can induce DNA damage in some endocrine‐resistant models (but not MCF‐7/LTED cells) and/or an unfolded protein response in others (but not HCC‐1428/LTED cells) [16, 18]. Understanding the molecular features that dictate different anticancer responses to estrogen would not only provide mechanistic understanding but also offer potential biomarkers and companion treatment strategies to improve efficacy.

## 5 Conclusions

Preclinical evidence showed entinostat had little efficacy against the treatment‐naïve MCF‐7/CA model of aromatase‐overexpressing ER^+^ BC; however, MCF‐7/CA xenografts with acquired resistance to the aromatase inhibitor (AI) letrozole were resensitized upon addition of entinostat [60]. Cotreatment of ER‐negative BC cells with the DNA methyltransferase inhibitor 5‐aza‐2′‐deoxycytidine plus the HDACi trichostatin A induced ER expression and rendered cells sensitive to endocrine therapy [61], suggesting epigenetic drugs can induce functional ER positivity in BC cells. Entinostat can also reverse the epithelial‐to‐mesenchymal transition [62]. Entinostat was thus tested clinically in combination with AIs for endocrine therapy‐resistant BC, but mixed results [21, 22, 23] led to the discontinuation of its development in this setting. In contrast, our findings collectively suggest that combination treatment with E2 and HDACi is superior to single‐agent treatment, and efficacy is a result of changes in chromatin accessibility, transcription, DNA damage, and cell cycle stress. These data warrant consideration of HDACi as a companion treatment for estrogen therapy in the setting of endocrine‐resistant ER^+^ BC, providing a scenario for rational testing of an HDACi in a solid tumor.

## Conflict of interest

The authors declare no conflict of interest.

## Author contributions

Conceptualization, ALJT and TWM; methodology, ALJT, BK, PCM, AMR, JR, HL, OMW, FWK, ED, BCC, and TWM; formal analysis, ALJT, BK, PCM, AMR, JR, AW, HL, KAB, MKF, AEG, HY, OWM, ED, and JMJ; resources, BCC and TWM; data curation, ALJT, BK, PCM, JR and TWM; writing‐original draft, ALJT and TWM; writing‐review and editing, all authors; supervision TWM; project administration, TWM; funding acquisition, TWM All authors have read and agreed to the published version of the manuscript.

## Supporting information


**Fig. S1.** Mocetinostat induced histone 3 acetylation and suppressed growth most effectively in combination with E2. (A) Cells were treated with HD medium ± mocetinostat as indicated for 24 h, and lysates were analyzed by immunoblot. (B) Cells were seeded in triplicate in HD medium and treated ±1 nm E2 ± mocetinostat for 28 days. Bars represent mean ± SD. Results shown are representative of 3 independent experiments. **P ≤ 0.01, ****P ≤ 0.0001 by Bonferroni‐adjusted *post hoc* test compared to respective ‘0 nM Mocetino’ group unless otherwise indicated. ns: not significant.
**Fig. S2.** Entinostat modulated hormone‐independent and E2‐induced expression of ER target genes. RT‐qPCR analysis of ER target genes (*AREG, PDZK1, TFF1*) in (A) HCC‐1428, (B) HCC‐1428/LTED, (C) MDA‐MB‐415/Luc, and (D) MDA‐MB‐415/ESR1 cells treated with HD medium ±1 nm E2 ± 500 nm entinostat. Expression values of the indicated genes were normalized to b‐actin mRNA (*ACTB*). Data are presented mean of triplicates ± SD *P ≤ 0.05, **P ≤ 0.01, ***P ≤ 0.001, ****P ≤ 0.0001 by Bonferroni‐adjusted *post hoc* test compared to respective control groups unless otherwise indicated. ns: not significant.
**Fig. S3.** Principal Components Analysis of RNA‐seq samples shows the major variables are cell type and treatment group. Treatment conditions are outlined in Fig. 3A. All groups had triplicate samples.
**Fig. S4.** Combined E2 and HDACi induce unique gene expression profiles. (A) Overlap analysis of differentially expressed (|log_2_ FC| ≥ 1 and *P* ≤ 0.05) genes shared between cell lines treated with 1 nm E2 as in Fig. 3A. Numbers of genes are indicated in bubbles. (B) Overlap analysis of combined up‐ and down‐regulated genes (relative to vehicle) identified under the indicated treatment conditions. Entinostat treatment lasted for the duration of 1, 3, or 7 days.
**Fig. S5.** Transcriptional upregulation induced by entinostat is maintained up to 7 days. Volcano plots of differentially expressed (|log_2_ FC| ≥ 1 and *P* ≤ 0.05) genes after 3 or 7 days of entinostat treatment and 1 day of E2 treatment as in Fig. 3A. Panel (A) shows single‐agent entinostat vs. vehicle. Panel (B) shows combination E2/entinostat vs. vehicle.
**Fig. S6.** Low‐dose entinostat did not alter cell cycle, replication, or DNA damage. HCC‐1428/LTED cells in HD medium were treated ±1 nm E2 ± 150 nm entinostat for 1 day. BrdU was spiked into the media 3 h prior to cell harvest. Cells were immunostained for BrdU, cleaved PARP, and yH2AX and counterstained with DAPI for flow cytometry analysis. (A) Cell cycle phase as inferred by DAPI signal. (B) Proportions of replicating cells. (C) Levels of DNA damage (γH2AX signal) in replicating cells shown as mean fluorescence intensity (MFI) ± SD. Cells that stained positively for cleaved PARP were excluded from (C). Data are presented as triplicate populations of ≥ 10 000 cells and shown as mean ± SD. Data were analyzed by Bonferroni‐adjusted *post hoc* test compared to respective control groups unless otherwise indicated. ns: not significant.
**Fig. S7.** Replicating cells showed more DNA damage than non‐replicating cells. HCC‐1428/LTED cells were treated with HD medium ±1 nm E2 ± 500 nm entinostat as indicated in triplicate. BrdU was spiked into media 3 h prior to cell harvest. Cells were immunostained for BrdU, cleaved PARP, and γH2AX with DAPI counterstain and analyzed by flow cytometry as in Fig. 6. Shown here is mean fluorescence intensity (MFI) of γH2AX as a measure of DNA damage in replicating (BrdU+) cells vs. the total cell population in each sample. Horizontal bars indicate group median. Results shown are representative of 3 independent experiments.
**Fig. S8.** Individual tumor growth curves for *in vivo* studies. Each graph depicts the volumes of individual tumors within each treatment group from Fig. 7A.
**Fig. S9.** Mocetinostat resensitizes resistant tumors to E2. Mice bearing WHIM16 tumors previously treated with E2 ± entinostat with recurrent tumors (that regrew during long‐term E2 treatment) were reimplanted with a fresh s.c. E2 pellet and randomized to treatment with mocetinostat (*n* = 4) or vehicle (*n* = 6). Tumor growth curves are shown as mean + SEM. ****P < 0.001 by linear mixed modeling of raw tumor volumes.
**Fig. S10.** RT‐qPCR analysis of ER target genes in xenografts. (A) Ovx mice bearing CTG‐3346 tumors were treated ± entinostat ± E2 for 3 days. (B) Ovx mice bearing WHIM16 tumors treated with vehicle or entinostat for 14 days were then co‐treated ± E2 for 3 days. In all cases, tumors were harvested at 4 h after the final drug treatment. RNA was harvested from frozen tumor fragments, and RT‐qPCR was performed. Expression values of the indicated genes were normalized to b‐actin mRNA (*ACTB*). Data are shown as mean of triplicate tumors + SD. **P ≤ 0.01, ***P ≤ 0.001, ****P ≤ 0.0001 by Bonferroni‐adjusted *post hoc* test compared to control group unless otherwise indicated. ns: not significant. <LD: below the limit of detection.
**Fig. S11.** E2 induced proliferation and DNA damage in CTG‐3346 tumors. Ovx mice bearing CTG‐3346 tumors were treated ± entinostat ± E2 for 3 days. On Day 3, mice were treated ± drug, and 1 h later mice were injected with EdU; tumors were harvested at 3 h post‐BrdU (which is 4 h post‐drug). FFPE tumors (*n* = 3/group) were immunostained for EdU, γH2AX, and cleaved caspase 3 (CC3) and counterstained with DAPI. (A) Representative immunofluorescent images (scale bar: 20 μm). (B) Quantification of EdU signal shown as proportion of positive (replicating) cells (mean + SD). (C) Quantification of γH2AX signal shown as mean fluorescence intensity (MFI) in ≥ 1000 nuclei per tumor. Horizontal bars indicate means. ***P ≤ 0.001, ****P ≤ 0.0001 by Bonferroni‐adjusted *post hoc* test compared to respective control (in B) or EdU‐ and EdU^+^ groups (in C) unless otherwise indicated. ns: not significant.

**Table S1.** qPCR primer sequences.
**Table S2.** ER binding sites identified by CUT&RUN. The genomic location of peaks identified from CUT&RUN for ER binding called in each treatment condition are separated into columns.
**Table S3.** Motif enrichment analysis of ER binding sites. ER‐bound genomic regions identified by CUT&RUN in response to treatment with E2 +/− entinostat were analyzed for estrogen response elements.

## Data Availability

Data generated by this study are available upon request to the corresponding authors. ATAC‐seq, RNA‐seq, and CUT&RUN datasets are available at NCBI as BioProjects PRJNA1392085, PRJNA1392066, PRJNA1474005, and PRJNA1392623.
