# Supplementary material for: Pharmacological chromatin remodeling enhances response to estrogen therapy in ER + breast cancer
Source: Mol Oncol. 2026 Jul 17:10.1002/1878-0261.70307. Online ahead of print. doi: 10.1002/1878-0261.70307 (PMC13398958; doi:10.1002/1878-0261.70307)

**Johnson et al. – Supplementary Information**

**Fig. S1****: Mocetinostat induced histone 3 acetylation and suppressed growth most effectively in combination with E2.**  (A) Cells were treated with HD medium ± mocetinostat as indicated for 24 h, and lysates were analyzed by immunoblot. (B) Cells were seeded in triplicate in HD medium and treated ± 1 nM E2 ± mocetinostat for 28 d. Bars represent mean ± SD. Results shown are representative of 3 independent experiments. **p≤0.01, ****p≤0.0001 by Bonferroni-adjusted posthoc test compared to respective “0 nM Mocetino” group unless otherwise indicated. ns: not significant.

**
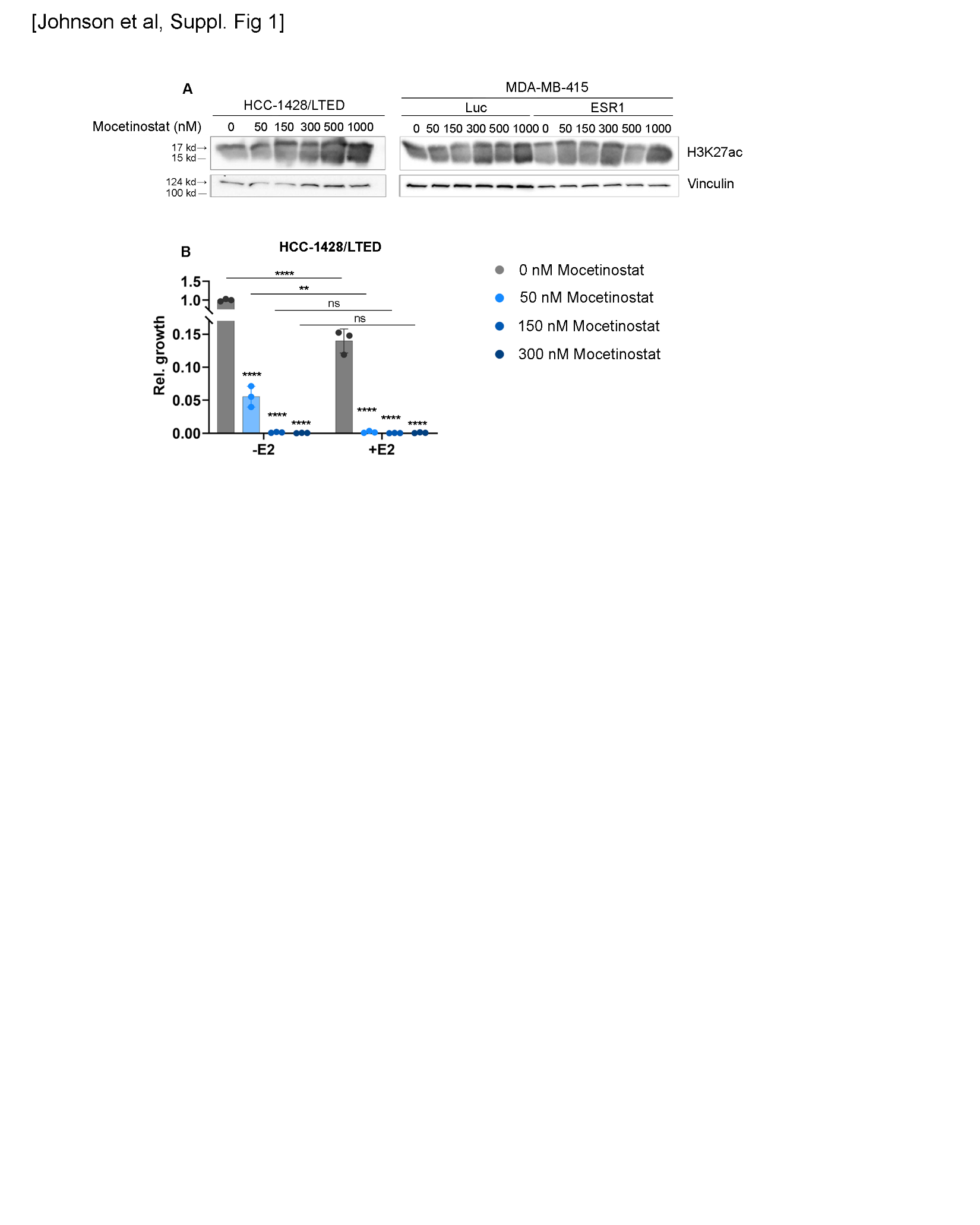
**

**Fig. S2: Entinostat modulated hormone-independent and E2-induced expression of ER target genes.** RT-qPCR analysis of ER target genes (*AREG, PDZK1, TFF1*) in (A) HCC-1428, (B) HCC-1428/LTED, (C) MDA-MB-415/Luc, and (D) MDA-MB-415/ESR1 cells treated with HD medium ± 1 nM E2 ± 500 nM entinostat. Expression values of the indicated genes were normalized to b-actin mRNA (*ACTB*). Data are presented mean of triplicates ± SD *p≤0.05, **p≤0.01, ***p≤0.001, ****p≤0.0001 by Bonferroni-adjusted posthoc test compared to respective control groups unless otherwise indicated. ns: not significant.


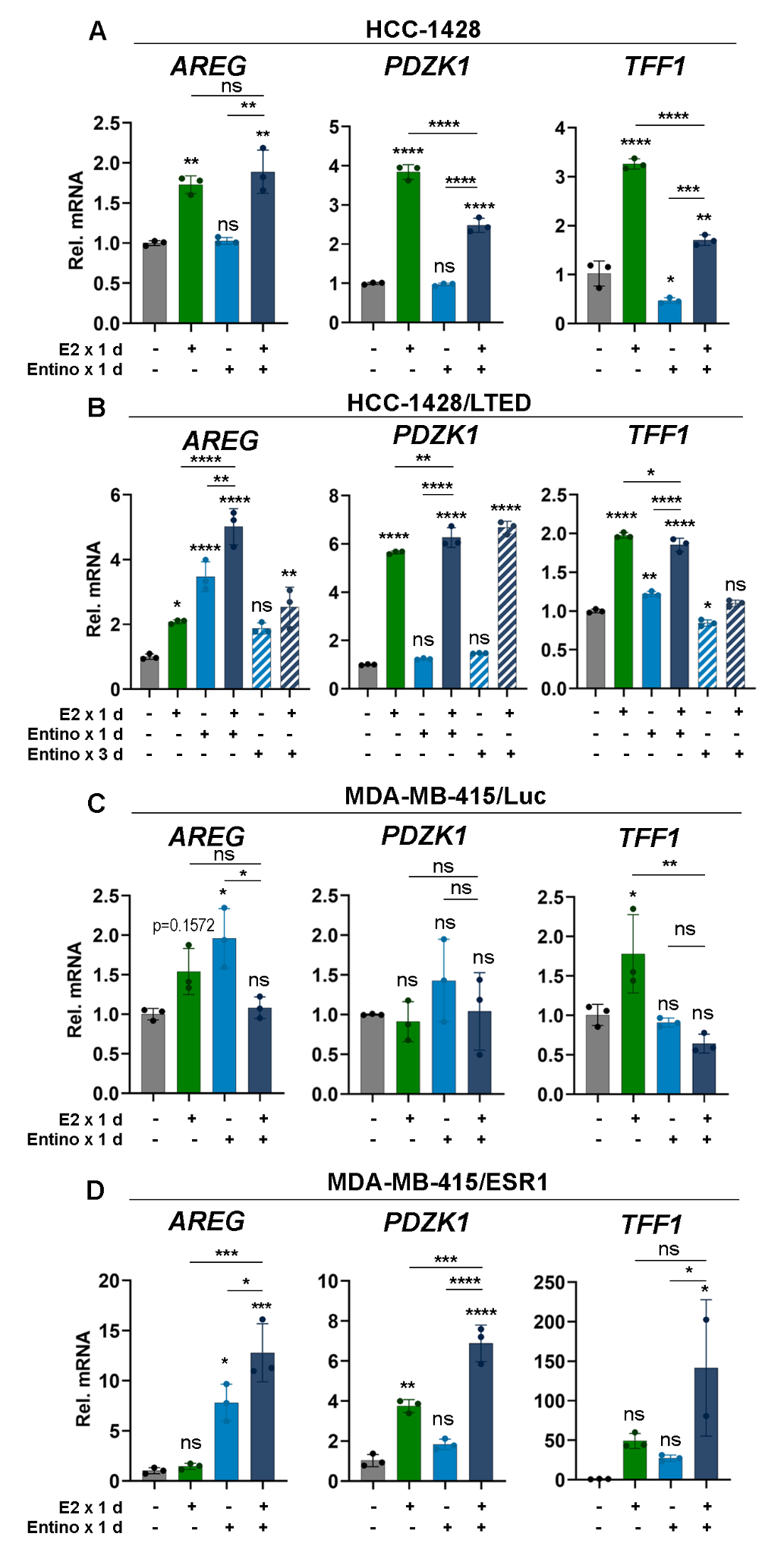


**Fig. S3: Principal Components Analysis of RNA-seq samples shows the major variables are cell type and treatment group.** Treatment conditions are outlined in Fig. 3A. All groups had triplicate samples.


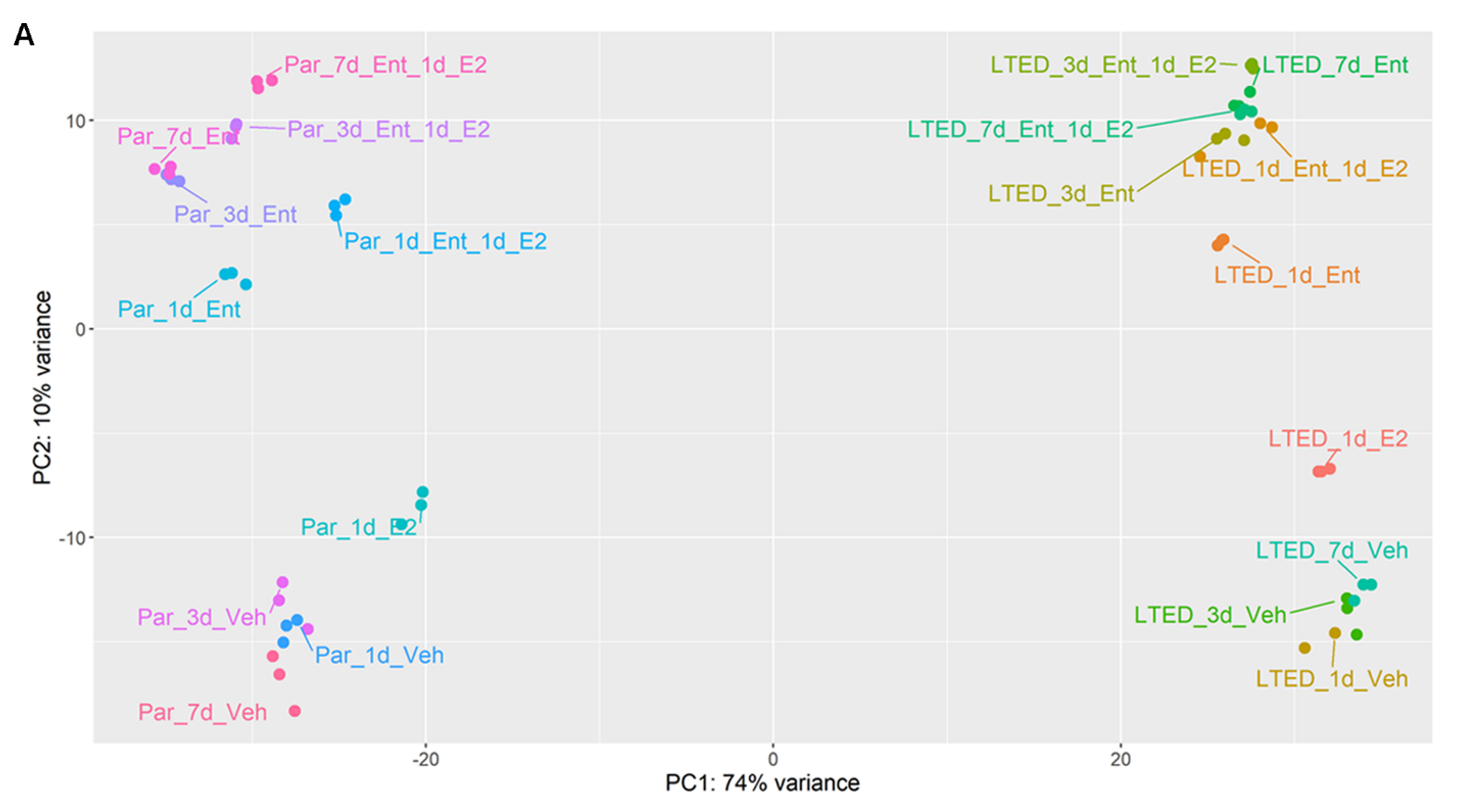


**Fig. S4: Combined E2 and HDACi induce unique gene expression profiles.** (A) Overlap analysis of differentially expressed (|log_2_ FC| ≥1 and p≤0.05) genes shared between cell lines treated with 1 nM E2 as in Fig. 3A. Numbers of genes are indicated in bubbles. (B) Overlap analysis of combined up- and down-regulated genes (relative to vehicle) identified under the indicated treatment conditions. Entinostat treatment lasted for the duration of 1, 3, or 7 d.


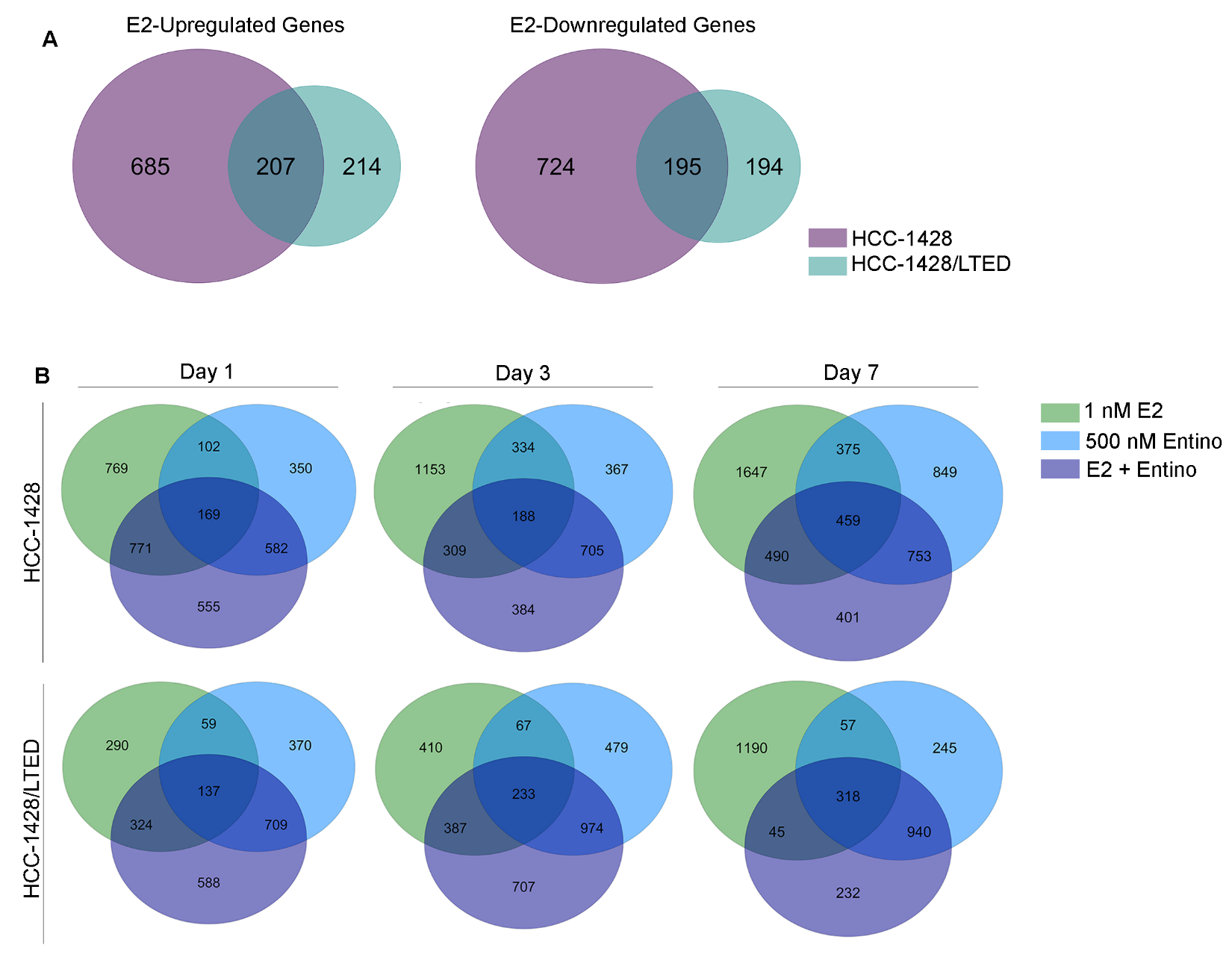


**Fig. S5: Transcriptional upregulation induced by entinostat is maintained up to 7 d.** Volcano plots of differentially expressed (|log_2_ FC| ≥1 and p≤0.05) genes after 3 or 7 d of entinostat treatment and 1 d of E2 treatment as in Fig. 3A. Panel (A) shows single-agent entinostat vs. vehicle. Panel (B) shows combination E2/entinostat vs. vehicle.


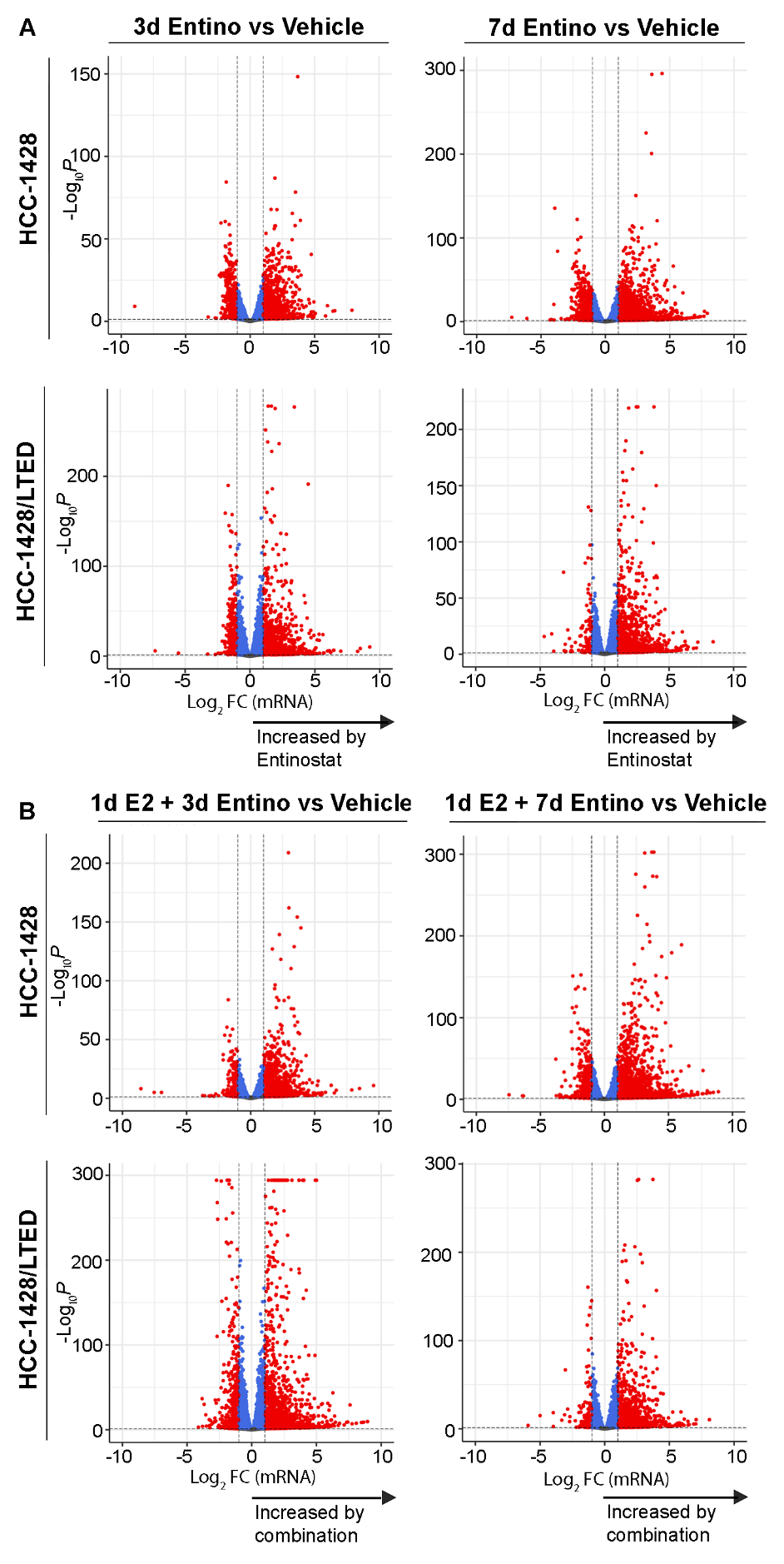


**Fig. S6: Low-dose entinostat did not alter cell cycle, replication, or DNA damage.** HCC-1428/LTED cells in HD medium were treated ± 1 nM E2 ± 150 nM entinostat for 1 d. BrdU was spiked into the media 3 h prior to cell harvest. Cells were immunostained for BrdU, cleaved PARP, and yH2AX and counterstained with DAPI for flow cytometry analysis. (A) Cell cycle phase as inferred by DAPI signal. (B) Proportions of replicating cells. (C) Levels of DNA damage (γH2AX signal) in replicating cells shown as mean fluorescence intensity (MFI) ± SD. Cells that stained positively for cleaved PARP were excluded from (C). Data are presented as triplicate populations of ≥ 10,000 cells and shown as mean ± SD. Data were analyzed by Bonferroni-adjusted posthoc test compared to respective control groups unless otherwise indicated. ns: not significant.


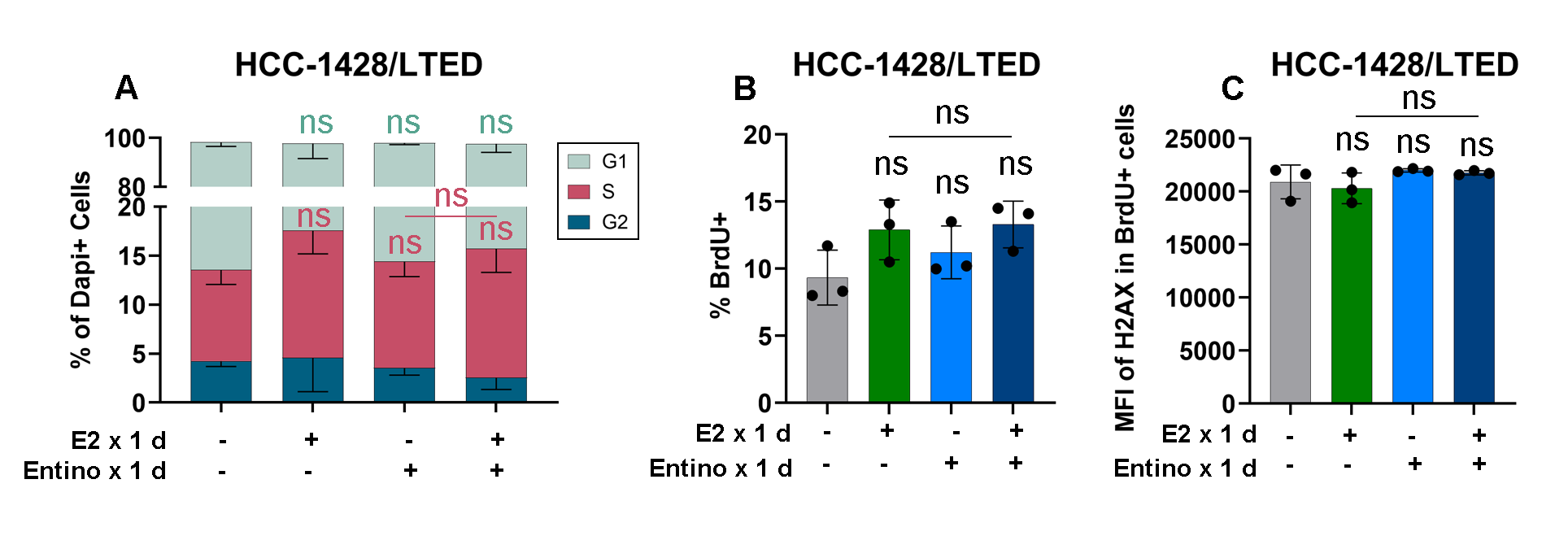


**Fig. S7: Replicating cells showed more DNA damage than non-replicating cells.** HCC-1428/LTED cells were treated with HD medium ± 1 nM E2 ± 500 nM entinostat as indicated in triplicate. BrdU was spiked into media 3 h prior to cell harvest. Cells were immunostained for BrdU, cleaved PARP, and γH2AX with DAPI counterstain and analyzed by flow cytometry as in Fig. 6. Shown here is mean fluorescence intensity (MFI) of γH2AX as a measure of DNA damage in replicating (BrdU+) cells vs. the total cell population in each sample. Horizontal bars indicate group median. Results shown are representative of 3 independent experiments.

**
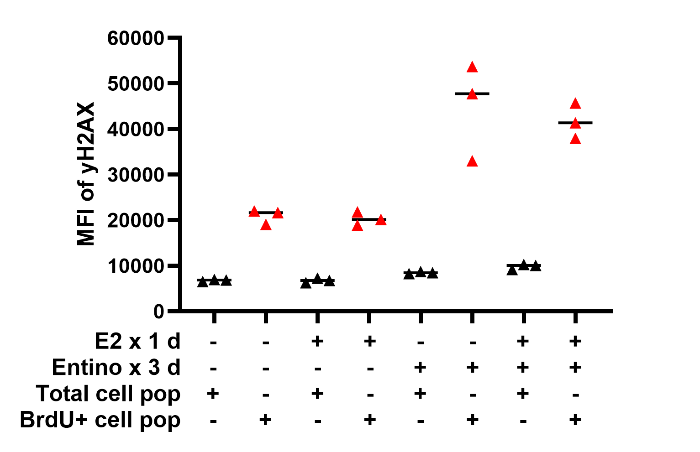
**

**Fig. S8: Individual tumor growth curves for *in vivo* studies.** Each graph depicts the volumes of individual tumors within each treatment group from Fig. 7A.


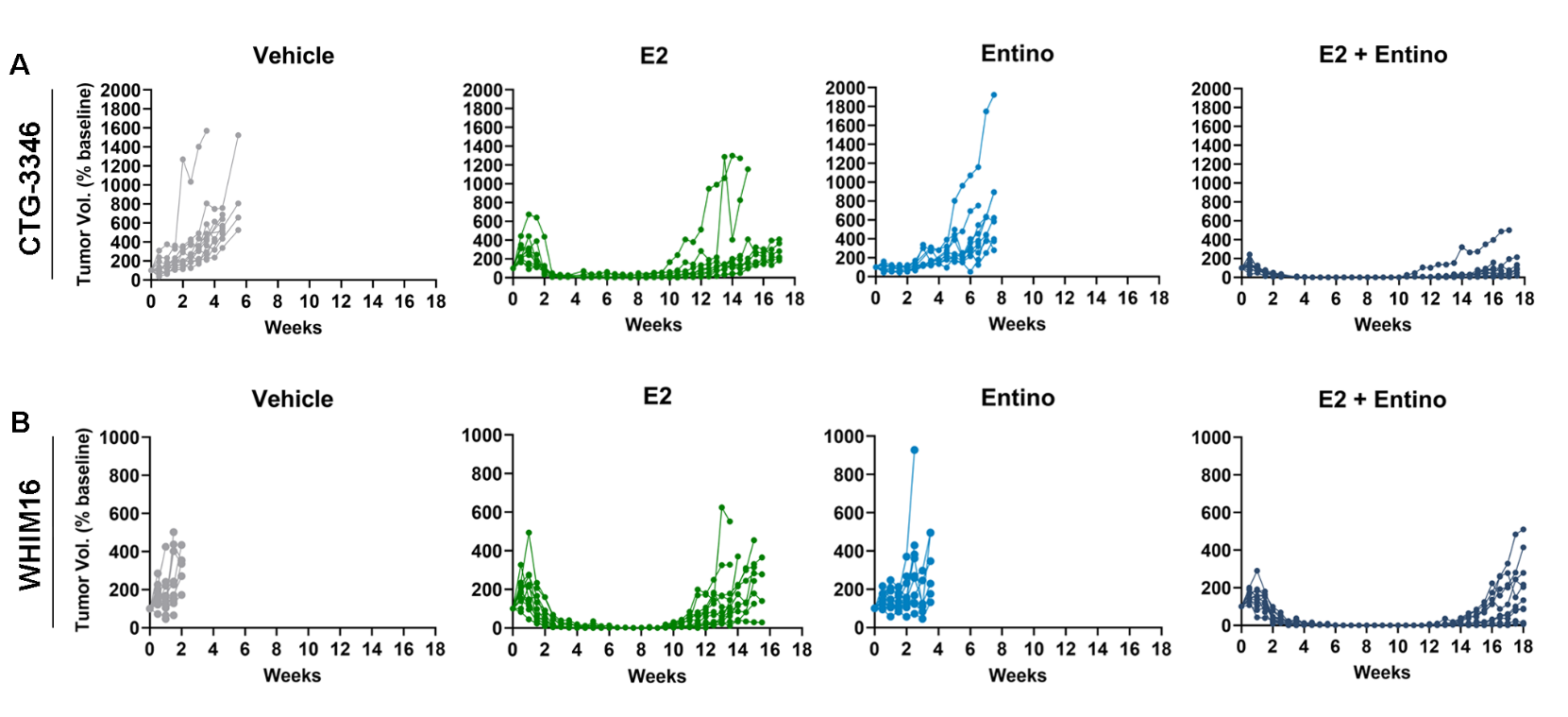


**Fig. S9: Mocetinostat resensitizes resistant tumors to E2.** Mice bearing WHIM16 tumors previously treated with E2 ± entinostat with recurrent tumors (that regrew during long-term E2 treatment) were reimplanted with a fresh s.c. E2 pellet and randomized to treatment with mocetinostat (n=4) or vehicle (n=6). Tumor growth curves are shown as mean + SEM. ****p<0.001 by linear mixed modeling of raw tumor volumes.


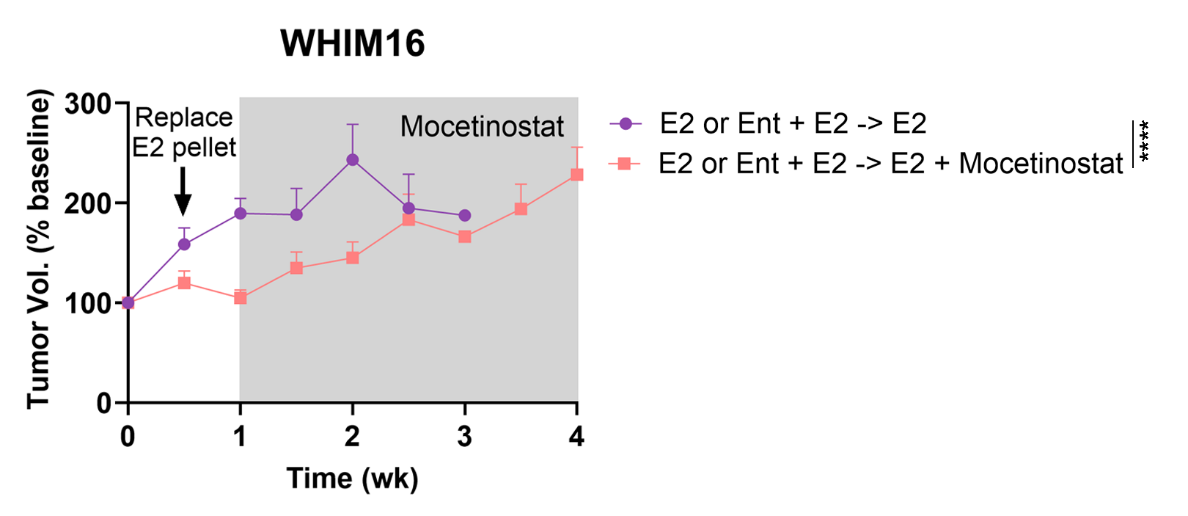


**Fig. S10: RT-qPCR analysis of ER target genes in xenografts.** (A) Ovx mice bearing CTG-3346 tumors were treated ± entinostat ± E2 for 3 d. (B) Ovx mice bearing WHIM16 tumors treated with vehicle or entinostat for 14 d were then co-treated ± E2 for 3 d. In all cases, tumors were harvested at 4 h after the final drug treatment. RNA was harvested from frozen tumor fragments, and RT-qPCR was performed. Expression values of the indicated genes were normalized to b-actin mRNA (*ACTB*). Data are shown as mean of triplicate tumors + SD. **p≤0.01, ***p≤0.001, ****p≤0.0001 by Bonferroni-adjusted posthoc test compared to control group unless otherwise indicated. ns: not significant. <LD: below the limit of detection.


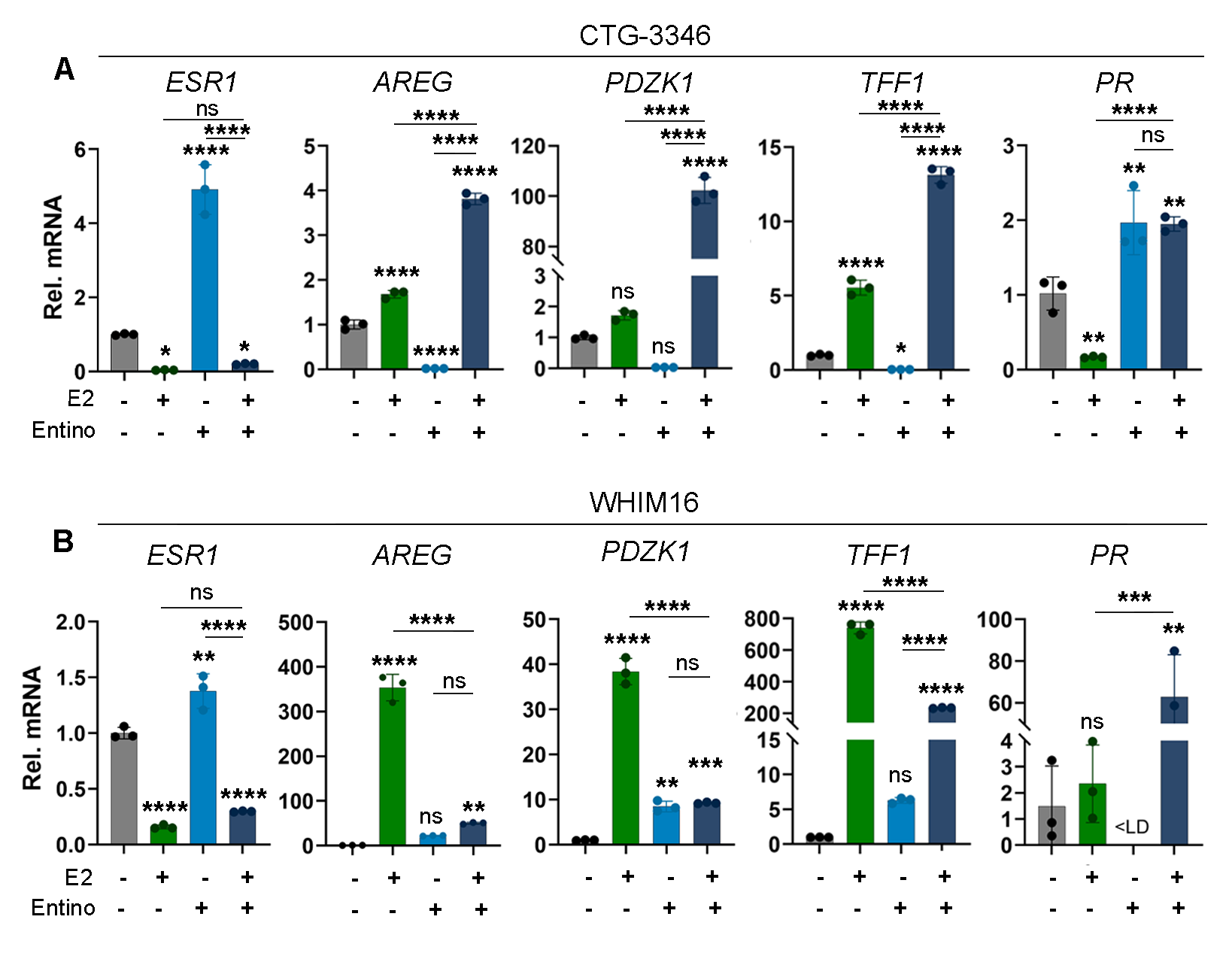


**Fig. S11: E2 induced proliferation and DNA damage in CTG-3346 tumors.** Ovx mice bearing CTG-3346 tumors were treated ± entinostat ± E2 for 3 d. On Day 3, mice were treated ± drug, and 1 h later mice were injected with EdU; tumors were harvested at 3 h post-BrdU (which is 4 h post-drug). FFPE tumors (n=3/group) were immunostained for EdU, γH2AX, and cleaved caspase 3 (CC3) and counterstained with DAPI. (A) Representative immunofluorescent images (scale bar: 20 um). (B) Quantification of EdU signal shown as proportion of positive (replicating) cells (mean + SD). (C) Quantification of γH2AX signal shown as mean fluorescence intensity (MFI) in ≥1,000 nuclei/tumor. Horizontal bars indicate means. ***p≤0.001, ****p≤0.0001 by Bonferroni-adjusted posthoc test compared to respective control (in B) or EdU- and EdU+ groups (in C) unless otherwise indicated. ns: not significant.


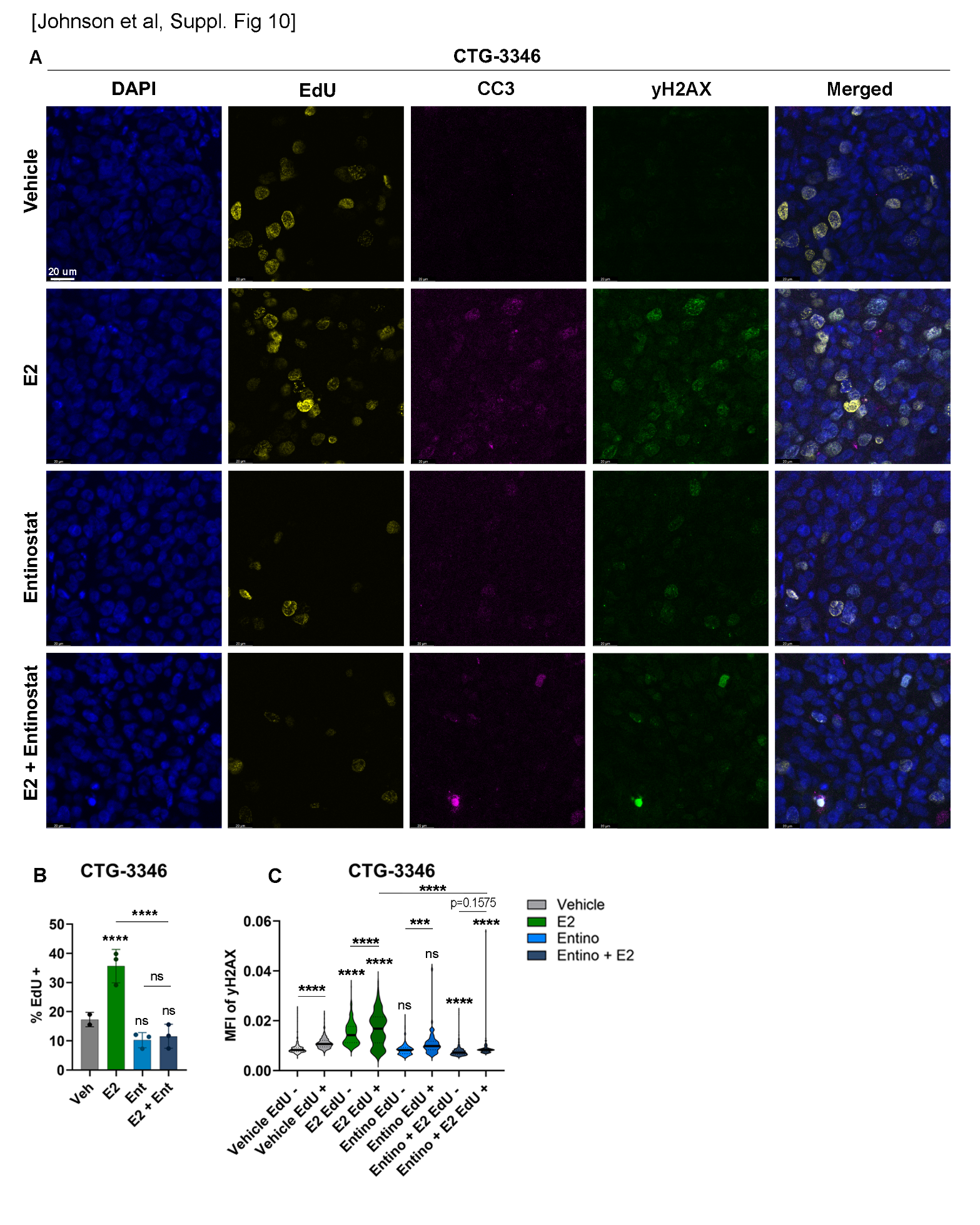

Supplement: Supplementary file 1 — Fig. S1. Mocetinostat induced histone 3 acetylation and suppressed growth most effectively in combination with E2. (A) Cells were treated with HD medium ± mocetinostat as indicated for 24 h, and lysates were analyzed by immunoblot. (B) Cells were seeded in triplicate in HD medium and treated ±1 nm E2 ± mocetinostat for 28 days. Bars represent mean ± SD. Results shown are representative of 3 independent experiments. **P ≤ 0.01, ****P ≤ 0.0001 by Bonferroni‐adjusted post hoc test compared to respective ‘0 nM Mocetino’ group unless otherwise indicated. ns: not significant. Fig. S2. Entinostat modulated hormone‐independent and E2‐induced expression of ER target genes. RT‐qPCR analysis of ER target genes (AREG, PDZK1, TFF1) in (A) HCC‐1428, (B) HCC‐1428/LTED, (C) MDA‐MB‐415/Luc, and (D) MDA‐MB‐415/ESR1 cells treated with HD medium ±1 nm E2 ± 500 nm entinostat. Expression values of the indicated genes were normalized to b‐actin mRNA (ACTB). Data are presented mean of triplicates ± SD *P ≤ 0.05, **P ≤ 0.01, ***P ≤ 0.001, ****P ≤ 0.0001 by Bonferroni‐adjusted post hoc test compared to respective control groups unless otherwise indicated. ns: not significant. Fig. S3. Principal Components Analysis of RNA‐seq samples shows the major variables are cell type and treatment group. Treatment conditions are outlined in Fig. 3A. All groups had triplicate samples. Fig. S4. Combined E2 and HDACi induce unique gene expression profiles. (A) Overlap analysis of differentially expressed (|log2 FC| ≥ 1 and P ≤ 0.05) genes shared between cell lines treated with 1 nm E2 as in Fig. 3A. Numbers of genes are indicated in bubbles. (B) Overlap analysis of combined up‐ and down‐regulated genes (relative to vehicle) identified under the indicated treatment conditions. Entinostat treatment lasted for the duration of 1, 3, or 7 days. Fig. S5. Transcriptional upregulation induced by entinostat is maintained up to 7 days. Volcano plots of differentially expressed (|log2 FC| ≥ 1 and P ≤ 0.05) genes afte [file MOL2-9999-0-s001.docx]
